# Engineering Ni-Silicide Nanocontacts for 3D Silicon Devices via Geometrical Confinement Control

**DOI:** 10.1021/acsnano.5c07195

**Published:** 2025-07-31

**Authors:** Jonas Müller, Remi Demoulin, Leonardo Cancellara, Fuccio Cristiano, Guilhem Larrieu

**Affiliations:** LAAS-CNRS, CNRS, 54928 University of Toulouse, Toulouse 31031, France

**Keywords:** Ni-silicide, nanowires, nanosheets, geometrical confinement, advanced nanoelectronics

## Abstract

Nanoscale Ni-silicide alloys are critical components for future generations of 3D electronic devices based on active Si nanostructures, with applications in nanoelectronics, energy conversion, and sensing. This study investigates how geometrical confinement in such nanostructures influences diffusion-driven silicidation, ultimately determining the alloy formation sequence, phase composition, and volumetric expansion. The silicidation of controlled Ni volumes is investigated on vertical silicon nanowires (NW) and nanosheets (NS) under various annealing conditions. The silicide phases and interface morphologies are characterized using high-resolution (scanning) transmission electron microscopy (HR-TEM, HR-STEM), energy-dispersive X-ray spectroscopy (EDX), and four-dimensional scanning transmission electron microscopy (4D-STEM) for nanoscale Ni–Si phase mapping. Under conditions of strong geometric confinement, NiSi_2_ is observed to form with faceted, prism-like morphologies aligned with Si (111) planes, features not typically present in planar or bulk samples. This anisotropic growth is associated with preferential Ni diffusion along nanostructure surfaces and limited Si counter-diffusion through the silicide. The resulting NiSi_2_ interfaces are structurally distinct and may contribute to reduced contact resistance in both p-type and n-type silicon nanostructures, supporting their integration in 3D device architectures.

## Graphical abstract

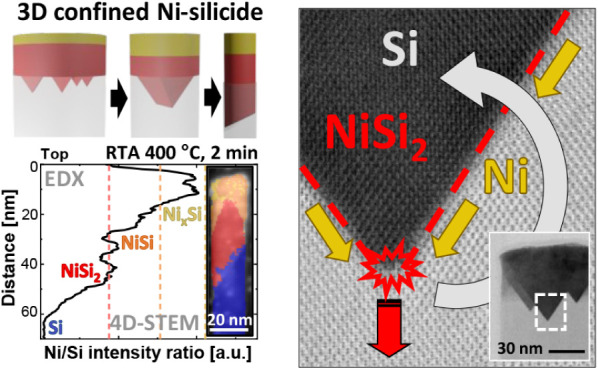

## Introduction

Nickel silicide (NiSi) is a key material in modern semiconductor technology,^1,2^ valued for its low resistivity, sufficient thermal stability under low-temperature processing conditions, and compatibility with CMOS processes. Over the past decade, NiSi has become a standard in advanced contact engineering, enabling continued device miniaturization and performance scaling in state-of-the-art CMOS architectures.^3^ As semiconductor devices evolve toward complex three-dimensional (3D) integration, the formation of nanoscale, low-resistance contacts within confined geometries presents new challenges. While the silicidation process is well established in planar microelectronics, its application to 3D nanostructures must account for strict thermal budgets, reduced material volumes, and the spatial constraints of ultrascaled, multilayered architectures. Among various silicides, NiSi is particularly attractive due to its relatively low formation temperature (400–500 °C), in contrast to high-temperature silicides such as CoSi_2_ and TiSi_2_.^4,5^ However, the Ni–Si system exhibits complex phase behavior, and the control of its transformation pathways becomes increasingly difficult at the nanoscale. In planar systems, the sequence of nickel silicide phase formation is well characterized (see Discussion 1 and Figure S1). However, this sequence can vary depending on process conditions such as annealing method^6^ (e.g., rapid thermal annealing (RTA)^1^ vs conventional furnace annealing^7–9^ ) and initial metal thickness.^10^ More critically, the influence of geometric confinement on Ni–Si silicidation—imposed by the nanoscale patterning of next-generation devices—remains poorly understood. Previous studies have primarily examined horizontal^11–14^ or vertical^15,16^ silicon nanowires (NW) with large, continuous Ni reservoirs^17,18^ or point contacts,^19–22^ often involving different crystallographic directions.^23^ In these systems, the metal reservoir can be considered effectively infinite relative to the silicon volume, and metal diffusion occurs asymmetrically, typically from a single surface. As a result, the diffusion process lacks uniformity across nanostructures of varying sizes, complicating direct comparison.

In contrast, the approach taken in this work, depositing a defined Ni layer directly on top of vertical nanostructures, introduces a symmetrical and controllable geometry. The volume of Ni involved in the reaction is precisely limited and normalized by nanowire diameter or nanosheet width, ensuring consistent reaction conditions across different structure sizes. This enables, for the first time, a systematic and quantitative comparison of silicide formation in nanostructures with different dimensions under identical thermal conditions. Achieving such control is essential for guiding phase formation and contact expansion, both of which are critical for integrating silicide contacts in gate-all-around (GAA) transistors with nanostructured channels, an emerging architecture for future CMOS technologies.^24^

In this study, we present a comprehensive investigation of Ni–Si silicidation in vertical silicon nanostructures, including nanowires and nanosheets (NS) with lateral dimensions down to ∼ 10 nm, using rapid thermal annealing. We systematically examine the influence of nanostructure size and annealing temperature on phase evolution, alloy expansion, and contact geometry using high-resolution (scanning) transmission electron microscopy (HR-TEM, HR-STEM), and energy-dispersive X-ray spectroscopy (EDX). Additionally, we introduce a novel 4D-STEM phase mapping approach that enables nanoscale identification of silicide phases and interfacial faceting. Based on these findings, we propose a general reaction mechanism for Ni-silicide formation in highly confined structures, governed by rapid interstitial Ni diffusion and self-limited Si transport, and evaluate the implications of interface morphology on contact resistance in field-effect transistors with nanostructured channel.

## Results

### Faceted Nickel Silicide Growth in Smallest Nanostructures

Multiple samples consisting of nanowires with dimensions ranging from 13 to 105 nm in diameter and nanosheets with 20–105 nm in width were fabricated using a top-down approach on unintentionally boron-doped (5 × 10^15^ cm^– 3^) silicon (001) substrates. A detailed description of the fabrication process and characterization is provided in the Methods section. As shown in Figure 1a, Si nanostructures with a height of 200 nm were patterned via e-beam lithography and etched using highly vertical reactive ion etching (RIE) with fluorine chemistry. Plasma-induced surface damage was minimized through the growth and subsequent wet-chemical removal of a sacrificial oxide (diluted HF dipping), before a 10 nm nickel layer was deposited with high directivity using physical vapor deposition (PVD). The silicidation process was then activated by rapid thermal annealing (RTA) under forming gas for 2 min. Due to the high verticality of the nanostructures and the anisotropic nature of metal deposition, silicide formation was confined to the top of the nanostructures and the planar Si substrate, as confirmed by SEM (Figure 1a).

**Figure 1:**
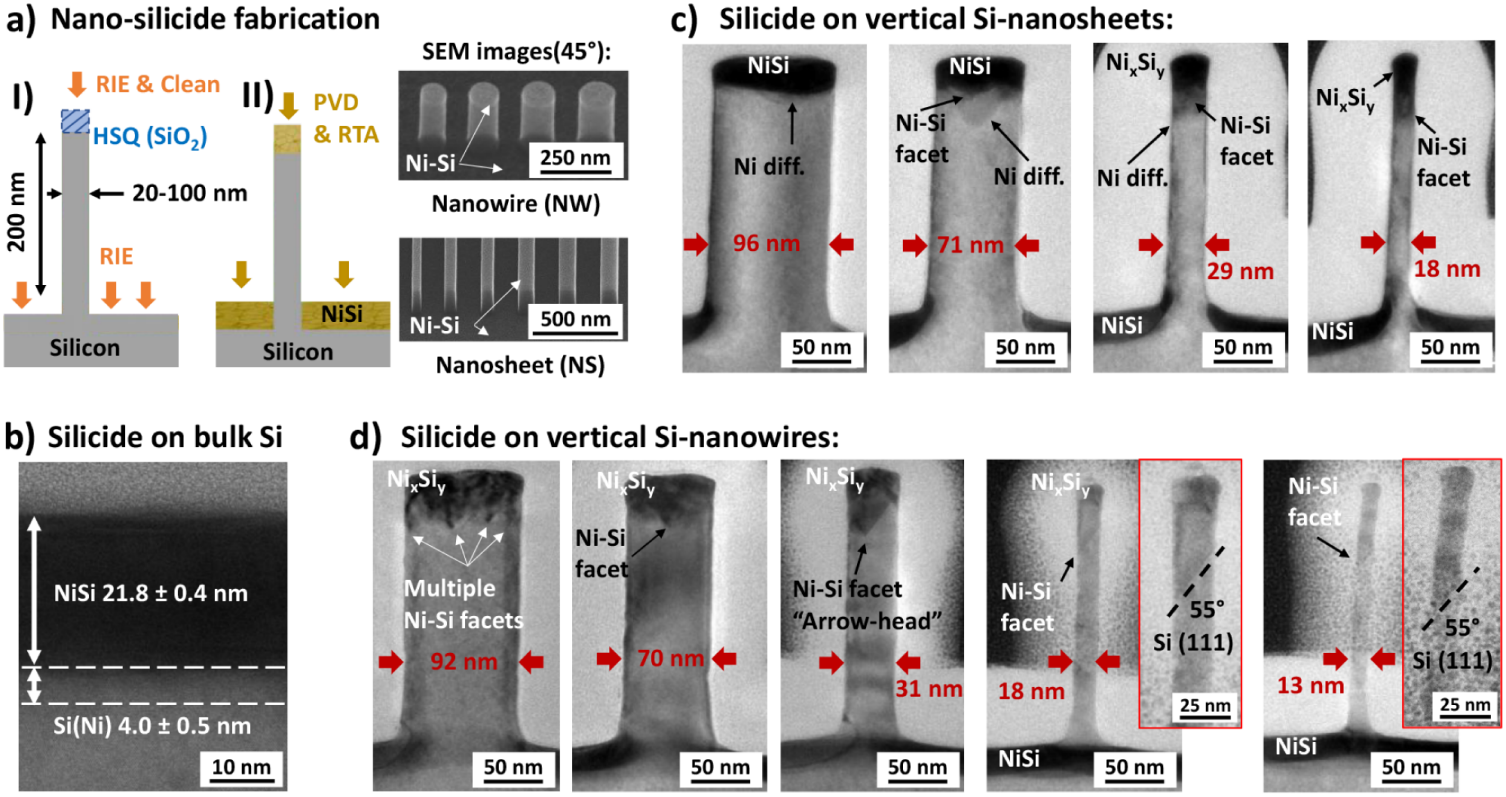
(a) Schematic of the top-down fabrication process and SEM images of vertical nanosheets (NS) and nanowires (NW) with silicide contacts: I) nanostructure etching by RIE using a HSQ mask; II) Ni deposition by PVD and silicidation by RTA after HSQ removal. (b–d) HR-TEM cross sections showing planar NiSi on bulk Si (b), and Ni–Si alloys on vertical NS (c) and NW (d) after 400 °C RTA. Large NS resemble bulk behavior, while small NS and all NWs, which exhibit faceted Ni_*x*_Si_*y*_ interfaces, show contact expansion beyond the expected NiSi thickness (∼22 nm). Facet number and depth increase with reduced structure size; all observed facets align ∼55° to the Si surface, consistent with the (111) plane.

The silicide formed on the nanostructures as well as on the bulk substrate is first investigated for RTA annealing at 400 °C, which is the reference temperature for the formation of NiSi on planar reference samples (see Figure S2; more information is provided in Discussion 2 in the Supporting Information). Figure 1b-d provide an overview of the contact morphology and interface formation in nanowires and nanosheets, compared to the bulk substrate. Under the given conditions, a homogeneous Ni–Si silicide layer with a thickness of 21.8 ± 0.4 nm forms on the surrounding planar Si substrate. The interface is mostly flat but features a nanometer-scale interfacial mixing layer where Ni has diffused into the silicon substrate (Figure 1b). Considering this additional interfacial mixing layer, the homogeneous silicide thickness aligns well with the expected NiSi thickness of 23.3 nm, as calculated from the deposited metal thickness and the known silicide expansion factor of 2.2 (see Table S1 for reference). While a similar silicide thickness is observed in large NS, the depth of Ni diffusion (Ni-diff.) appears to be greater at the center of the nanostructures (Figure 1c). As the NS width decreases, the alloy expands, increasing the depth of the contact interface while simultaneously developing a nonflat, faceted interface in the smallest NS. Similarly, faceted interfaces and increased alloy thicknesses are observed across all NW dimensions. In larger nanowires, multiple nucleation sites lead to the formation of faceted protrusions, while the number of facets decreases with smaller NW diameters. Eventually, the interface evolves into either a triangular “arrowhead” shape or a single remaining facet. In all cases, the faceted Ni_*x*_Si_*y*_ interfaces form an angle of 51–55° to the Si surface, corresponding to the (111) planes of the silicon substrate, as indicated in Figure 1d.

### Temperature-Based Study of Ni-Silicide Formation in NW/NS

Similar faceted growth has already been reported in the literature for silicides on both planar substrates and nanostructures under various conditions. For instance, pyramidal structures originating from epitaxial NiSi_2_ have been observed in ultrathin films^25^ and thin films with oxide interlayers.^26^ However, these structures were not thermally stable and transformed into NiSi at temperatures above 400 °C under conventional furnace annealing. For RTA annealing, NiSi_2_ facets have been reported to be stable but were only observed at higher temperatures of 450 °C in both planar and nanowire structures.^11,12,27,28^ In cases where the Ni reservoir was limited,^16^ RTA at 500 °C for 4 min resulted in similar NiSi_2_ facet growth, but only for larger nanowires with diameters *d* > 100 nm, while smaller NWs exhibited a NiSi interface instead. Interestingly, a recent in situ study by Hou et al.^29^ demonstrated the possibility of pyramidal Ni-rich structures, such as Ni_2_Si, forming at low temperatures. These structures may transform into NiSi while preserving their faceted interfaces. To fully understand the observed silicide formation and the sequential transformation under high geometric constraints in nanostructures of different sizes and geometries, we compare the Ni-silicide expansion and interface evolution in arrays of nanosheets and nanowires across annealing temperatures of 300 °C, 400 °C, and 500 °C. The fabricated samples are analyzed using HR-TEM, and Figure 2 presents a comparison of the homogeneous main silicide layer thickness and the additional penetration depth of the faceted interface for NS and NW after 2 min annealing at each temperature. Without geometric confinement, planar silicide formation on silicon results in a homogeneous silicide layer under all three annealing conditions. EDX analysis confirms that at 400 °C, the silicide consists purely of NiSi, with a thickness of 21–22 nm (Figures S3 and S4). At 300 °C, despite an identical thickness, the presence of Ni_2_Si surface grains indicates an incomplete transformation. At 500 °C, the silicide layer slightly expands, forming an interfacial Ni_*x*_Si_*y*_ layer and voids near the surface. In all cases, a thin mixing layer is observed, where Ni atoms diffuse into the Si substrate without forming silicide. Similar interfaces appear in large NS representing nanostructures with weak 1D confinement. However, when the silicide reaction occurs within a sufficiently small reaction volume, faceted Ni–Si growth is promoted, reducing the homogeneous silicide volume. In NS, this transition happens below a critical dimension of 70–80 nm. For the high geometrical 2D-confinement in nanowires, faceted silicide interfaces are present for all observed nanowire diameters, suggesting a critical dimension exceeding 100 nm.

**Figure 2:**
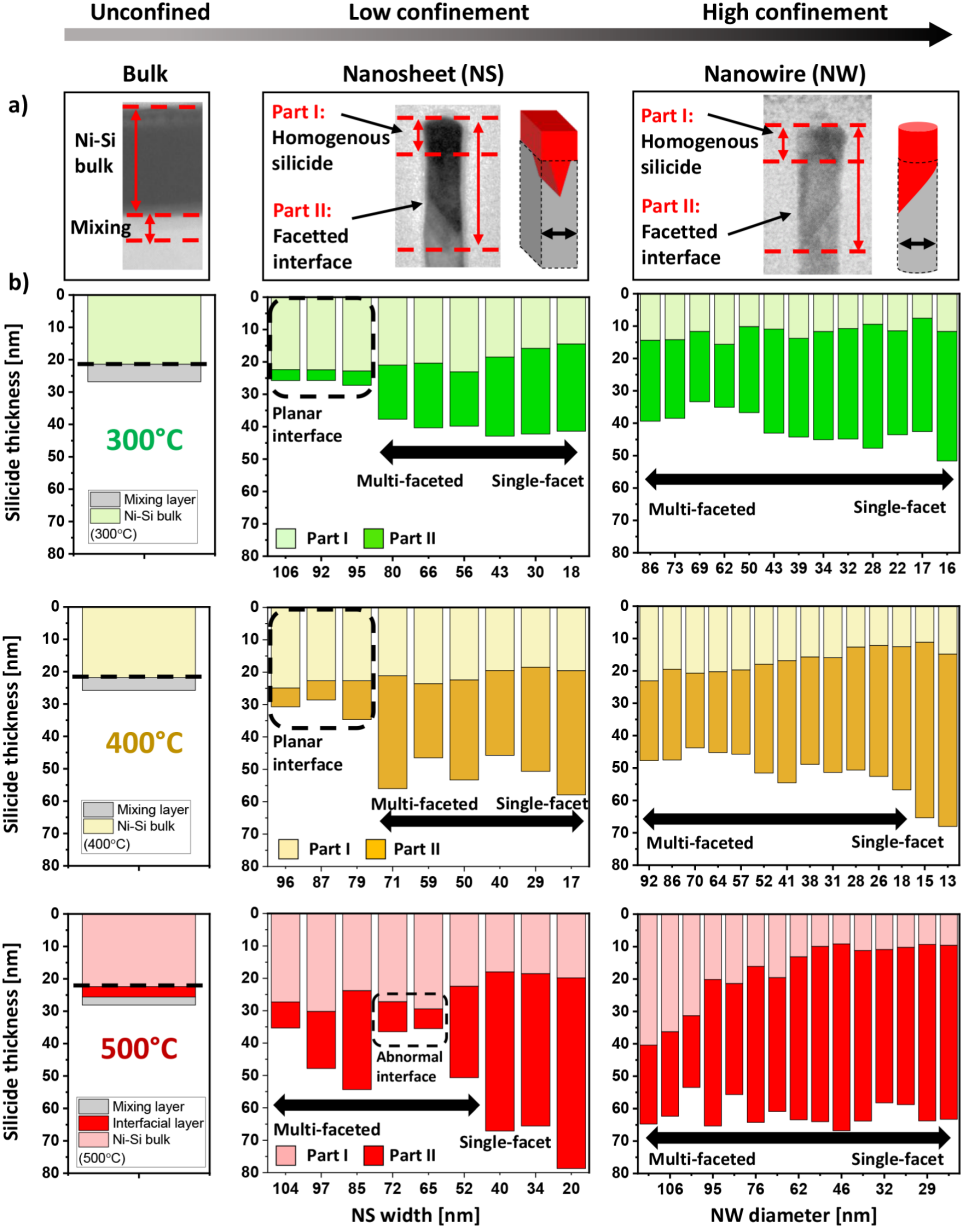
Characterization of Ni-silicide expansion by TEM observation for Ni–Si alloys on planar Si and differently sized vertical Si nanosheets and nanowires (a). The initial nickel thickness is 10 nm for all samples and silicidation has been carried out by RTA at different temperatures during 2 min at 300, 400, and 500 °C, highlighted in (b) as green, yellow and red, respectively. Averaged silicide thickness measurements of the observed homogeneous silicide layer (Part I) and the extended faceted-interface (Part II) have been measured based on intensity profiles of recorded TEM images.

For both NS and NW, the faceted phase thickens as their dimensions decrease, partially due to the transition from a multifaceted to a single-facet interface. Larger nanostructures support multiple nucleation sites across the interface, while smaller ones, with limited Si volume, allow only a single nucleation site. This transition occurs irrespective of nanostructure geometry, as long as at least 1D confinement is present. Faceted Ni–Si growth scales with annealing temperature across different nanostructure geometries: from 25 to 45 nm at 300 °C, from 45 to 70 nm at 400 °C and from 63 to 65 nm at 500 °C.

To investigate the transformation under strong and weak 1*D*/2D confinement, selected NS/NW nanostructures with small (17–25 nm) and large (73–106 nm) dimensions were characterized by EDX under all three annealing conditions. Due to the limited Ni–Si volume in the smallest nanostructures, particularly nanowires, the signal-to-noise ratio in EDX measurements was too low for standard chemical quantification. Instead, raw Ni:Si intensity ratios obtained from EDX line scans were recorded and analyzed through comparison with calibrated data from bulk reference silicide layers representing different Ni–Si phases (see Supporting Information Discussion 4). These reference ratios were correlated with their respective quantified compositions (Figures S4–S6) and used to construct a conversion table (Figure S7). This table provides phase-specific intensity benchmarks, which are represented as horizontal reference lines in Figure 3 to support interpretation of the EDX data. High-Ni-content silicides (e.g., Ni_3_Si, Ni_31_Si_12_) are hereafter conservatively referred to as Ni_(*x*>2)_Si to reflect their uncertain stoichiometry within the Ni-rich regime. In large NS with low 1D-confinement (Figure 3a), a homogeneous Ni_2_Si layer forms at 300 °C, transforming into NiSi at 400 °C, where it remains stable up to 500 °C. At this stage, Ni accumulation at the NiSi/Si interface and a decrease in Ni density at the surface suggest the formation of an interfacial layer, clustering, and void formation, similar to planar Ni–Si systems. In small NS below the critical confinement threshold (Figure 3b), higher 1D confinement slows the silicide reaction. Initially at 300 °C, the homogeneous silicide phase is Ni-rich with a composition of Ni_**(** *x* **>2**)_Si, possibly corresponding to a mixture of finite volumes of Ni-rich silicide such as Ni_3_Si, Ni_31_Si_12_ and Ni_2_Si. This silicide layer directly transitions into the faceted interface (NiSi_*z*_). At higher temperatures, the fully transformed Ni_2_Si phase is positioned atop a mixed Ni_*x*_Si_*y*_ phase. This Ni_*x*_Si_*y*_ region transitions into the faceted interface with a variable composition (x < y), likely corresponding to a mixture of NiSi and NiSi_2_ domains. At even higher temperatures, the Ni-rich silicide transforms into Ni-poor NiSi_2_. The NiSi_*z*_ interface shows a decreasing Ni/Si gradient at all temperatures due to its shrinking volume.

**Figure 3:**
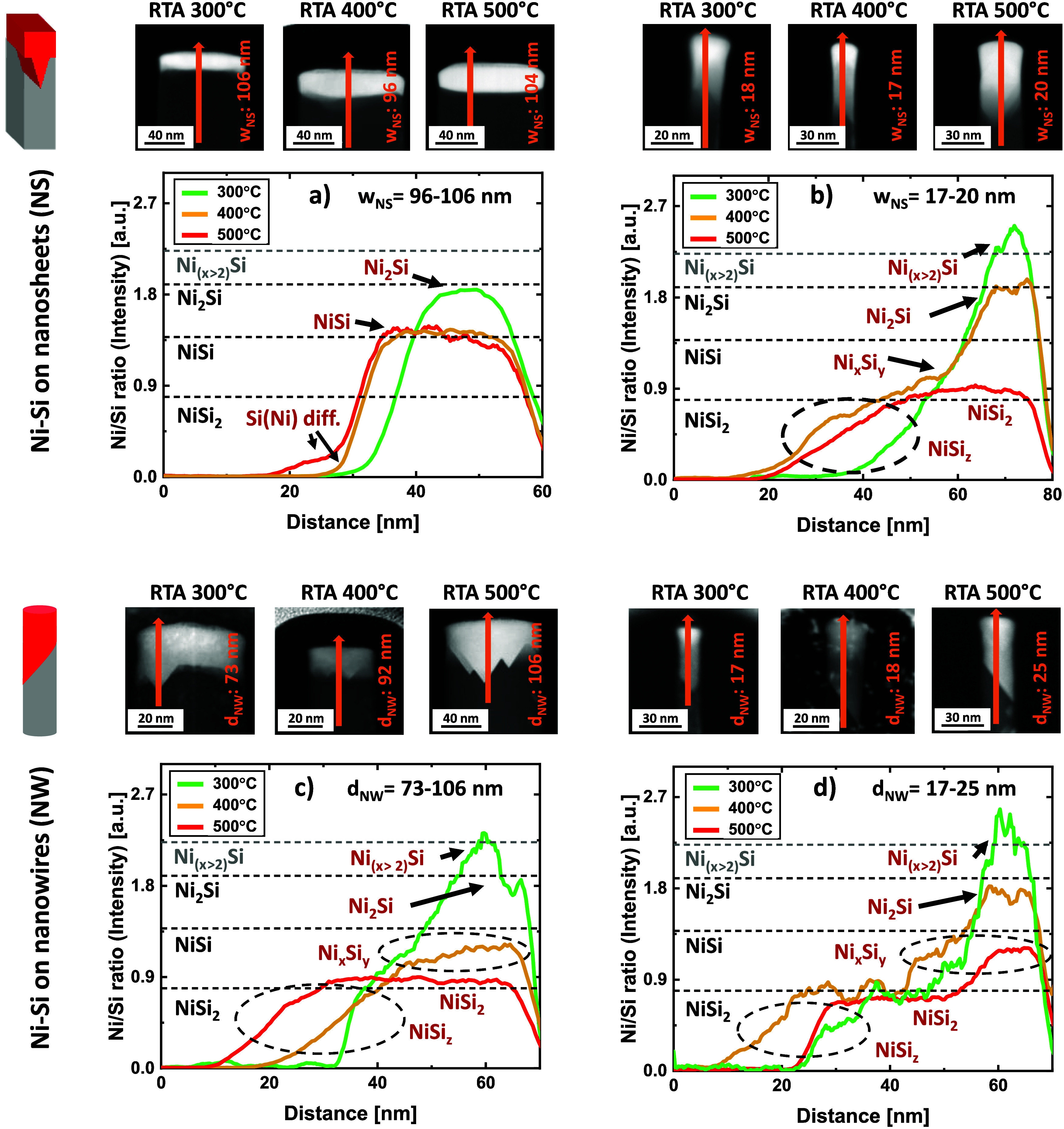
EDX line scans of selected nanosheets (NS) and nanowires (NW) with corresponding Ni/Si intensity ratios for large structures (a, c) and small structures (b, d). Line scans were taken along the orange arrows in the STEM-HAADF images. Identified Ni–Si phases are assigned based on reference Ni/Si ratios from bulk silicide layers (Figure S7), with phase-specific reference lines shown as dashed horizontals.

In large NWs (Figure 3c), where NiSi_*z*_ facets are already present due to 2D-confinement, the initial homogeneous Ni-rich Ni_**(** *x* **>2**)_Si layer transitions directly into the faceted interface at 300 °C. By 400 °C, the homogeneous silicide converts into Ni_*x*_Si_*y*_, with a varying composition from NiSi close to NiSi_2_. After 500 °C annealing, the silicide is fully transformed into NiSi_2_. The stabilization of Ni-rich silicide phases observed for small NS is even more pronounced in small NWs (Figure 3d), where Ni_*x*_Si_*y*_ phases appear at all temperatures and remain close to NiSi in composition. However, near the faceted NiSi_*z*_ interface, a large NiSi_2_ region forms regardless of temperature.

EDX analysis successfully identifies the homogeneous top silicide layers, including Ni-rich silicides Ni_**(** *x* **>2**)_Si (without identifying the exact phase or composition), Ni_2_Si, NiSi as well as NiSi_2_. Complementary phase identification has been carried out by Fast-Fourier-Transform (FFT) analysis, as presented in in Discussion 4 in the Supporting Information (Figures S8–S11), and identified a large volume of faceted NiSi_*z*_, obtained at high temperature, as NiSi_2_ (Figure S11). Since NiSi_*z*_ growth remains consistent across all temperatures and nanostructures with critically small reaction volumes, it is inferred that the facets in all observed structures correspond to NiSi_2_. However, at lower temperatures (300–400 °C), the faceted volume remains small compared to the surrounding Si matrix, raising the possibility of Ni-rich facets enveloped by Si, which could generate a similar EDX signature. As direct characterization of small NiSi_*z*_ volumes using EDX or FFT was not feasible, a novel 4D-STEM technique was developed to reliably identify minute silicide quantities within a Si matrix.

To confirm the presence of NiSi_2_ facets at lower temperatures, a Ni–Si phase map was calculated for a silicide nanoalloy annealed at 400 °C (Figure 4a). The sample was scanned with an electron beam to record corresponding diffraction patterns pixel by pixel using a 1 nm step size, creating a 4D data cube which is successively analyzed using the automated crystal orientation mapping (ACOM) presented in ref 30. A modified py4DSTEM^31,32^ procedure has been implemented comparing diffraction patterns pixel-by-pixel with a Ni–Si phase reference library.^33^ The algorithm calculates the orientation-maps for individual Ni–Si phases with nanoscale resolution (see also Figure S12a), assigning a correlation value to the best-match. Based on this value a multiphase map is generated, assigning to each pixel the phase with the highest correlation. ACOM enables precise differentiation between silicide phases of different crystal structures, such as the orthorhombic NiSi and cubic NiSi_2_, with just a manual calibration of the calculation’s parameters. However, similarities in crystal structures, such as for NiSi_2_ and Si both cubic, or Ni-rich alloys, may lead to artificial phase switching in the final multiphase map. To mitigate this, ACOM’s parameters were optimized using a genetic algorithm, providing as goal the minimization of the correlation value’s standard deviation for a region with known phase and orientation (i.e., the [110] Silicon base), furthermore excluding solutions that assigned silicon to the silicide regions. The Ni–Si phase map resulting from the ACOM with optimized parameters reveals a well-defined NiSi region underneath a Ni-rich surface layer in small nanostructures and confirms the complete absence of NiSi within the faceted protrusions, demonstrating that the interfacial phase present at all temperatures is indeed NiSi_2_. The Ni_3_Si phase has been suppressed in the final multiphase output to reduce the number of artifacts (see also Figure S12b), labeling Ni-rich areas that were not attributed to NiSi as Ni_*x*_Si.

**Figure 4:**
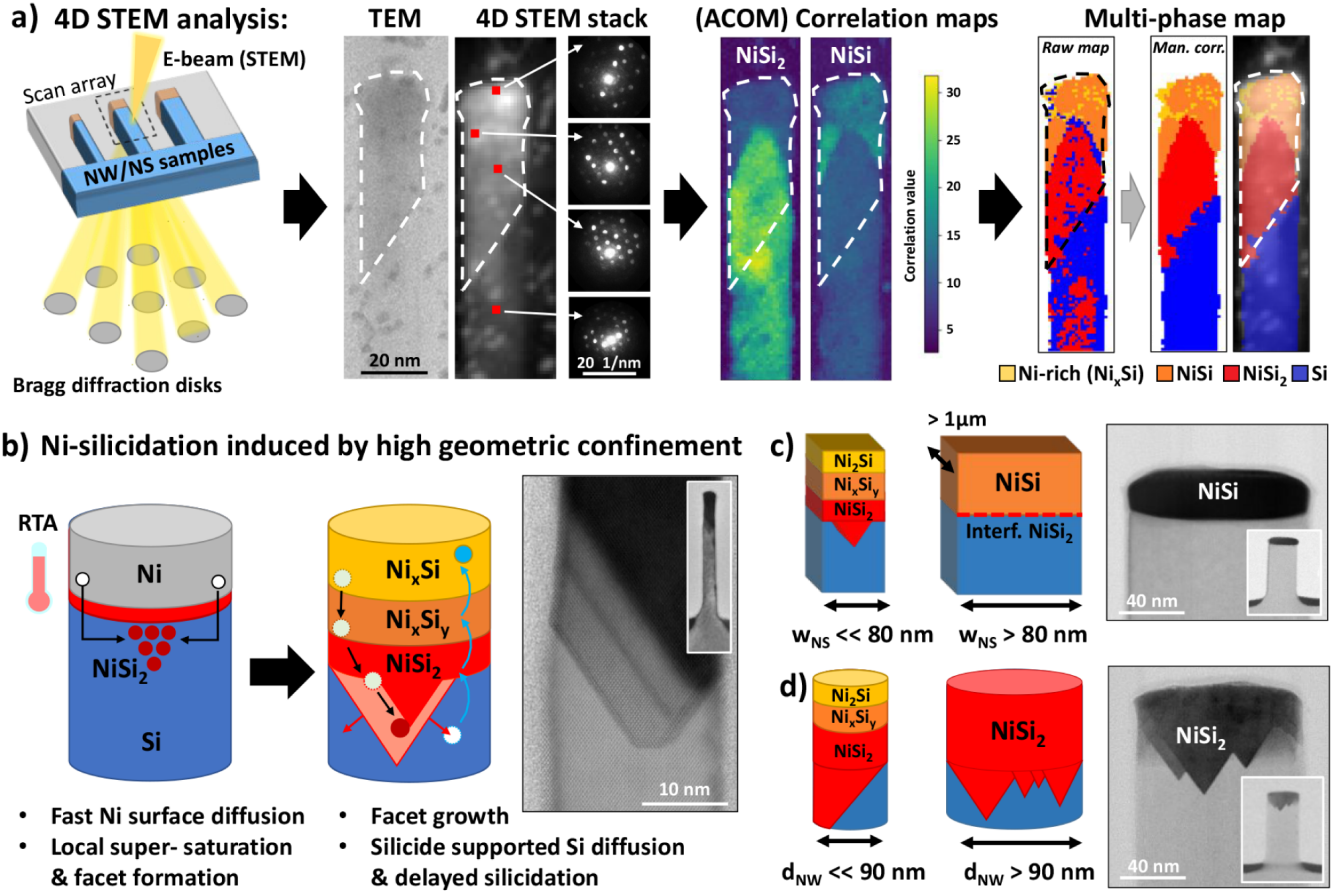
(a) 4D-STEM phase mapping used to identify faceted Ni–Si phases in confined nanostructures. A scanned STEM beam records diffraction patterns pixel by pixel, which are matched to reference data (Ni_3_Si, Ni_2_Si, NiSi, NiSi_2_, Si) using a genetic algorithm. Resulting correlation maps are shown here for NiSi and NiSi_2_, further correlation maps are presented in Figure S12. Despite overlap between similar structures (e.g., NiSi_2_ and Si), orthorhombic NiSi and cubic NiSi_2_ can be clearly distinguished. The final multiphase map confirms the faceted silicide protrusion as NiSi_2_ (red). A manual correction of phase-switching artifacts has been applied for visual clarity. (b) Schematic of the proposed silicidation process under high confinement, highlighting rapid Ni surface diffusion, initial NiSi_2_ formation, and self-retarded Si diffusion. (c, d) Geometric effects: in small NS and NWs, confinement limits Si diffusion, stabilizing Ni-rich phases; relaxing the dimensional confinement in larger NWs (d) allows full transformation into NiSi_2_, whereas the further reduction in geometrical confinement in large NS (c) permits NiSi formation, similar to planar substrates.

### Overview of the Ni–Si Diffusion Process under Geometrical Confinement

Using a similar diffusion picture to that proposed in ref 16, we present a schematic Ni-silicidation mechanism in nanostructures of varying dimensions on (001)-Si in Figure 4b (see Figure S13 for an extended illustration). After deposition, nickel begins to diffuse into the silicon substrate even at room temperature by occupying interstitial sites (Figure S8b). Upon annealing, these interstitial Ni atoms may form a thin mixing layer or an epitaxial NiSi_2_ layer, even at low temperatures such as 300 °C.^12,15,34^ Ni continuously diffuses from the Ni-layer into the silicon, driven by the nickel concentration gradient.^35^

In nanostructures, this interstitial diffusion is primarily channeled along the surfaces and interfaces—specifically, along the nanostructure’s sidewalls and the Ni–Si boundary.^36,37^ As Ni accumulates along these paths, local supersaturation can occur, leading to the precipitation of NiSi_2_. This typically happens at the center of small nanostructures^20,21^ or at multiple, randomly distributed sites in larger ones. The initial formation of faceted NiSi_2_ interfaces is driven by two main factors. First, interfacial stress reduction: facets aligned along the (111) planes reduce strain at the interface due to the minimal lattice mismatch between the cubic NiSi_2_ and Si (−0.4%),^38,39^ in contrast to much higher mismatches for orthorhombic NiSi (−62.1%, −5.0% or 2.7% depending on orientation). Second, the dominance of fast Ni diffusion over Si diffusion: at early stages, the silicidation process is primarily governed by the rapid supply of Ni atoms, which is strongly influenced by nanostructure size and geometry. Smaller nanostructures, with their high surface-to-volume ratio, enhance surface diffusion of Ni and promote the continued growth of faceted NiSi_2_ interfaces. In contrast, for larger nanostructures or near-planar geometries, the contribution of surface diffusion becomes negligible. As a result, a more homogeneous NiSi layer forms (Figure 4c), and any NiSi_2_ is limited to an atomically thin interfacial layer—or entirely suppressed in planar cases.

The growth of the NiSi_2_ phase also plays a critical role in controlling Si diffusion and, in turn, the transformation of the remaining Ni layer. As Ni continues to diffuse downward into the silicon, Si atoms diffuse upward through vacancies within the growing NiSi_2_ layer. In small nanostructures, the extended NiSi_2_ interface lengthens the diffusion path, thereby slowing the Si supply to the Ni-rich region. This self-limiting mechanism explains the stabilization of near-surface Ni-rich phases such as Ni_2_Si or potentially Ni_3_Si and Ni_31_Si_12_ in confined nanostructures, even when these phases have already transformed into NiSi or NiSi_2_ in planar substrates under the same RTA conditions. At sufficiently high temperatures or with longer annealing times, enough Si may diffuse through the NiSi_2_ layer to fully transform the Ni-rich phases into a homogeneous NiSi_2_ alloy (e.g., ref 15). This is analogous to the behavior in large nanowires, where the thinner, multifaceted NiSi_2_ interface allows more efficient Si diffusion (Figure 4d).

Overall, our results demonstrate that high geometric confinement in vertical nanostructures promotes the formation of NiSi_2_ at all observed temperatures down to 300 °C. The resulting NiSi_2_ interface not only defines the morphology of the contact but also modulates the local distribution of Ni-rich silicide phases during short-time annealing. Both Ni diffusion along the interface and Si counter-diffusion through the growing NiSi_2_ phase are strongly geometry-dependent, giving rise to the formation of distinctly faceted NiSi_2_ interfaces as a function of nanostructure size and dimensionality.

### Impact of Faceted NiSi_2_ Nanocontacts on Contact Resistance

We assess the impact of the observed faceted NiSi_2_ interfaces on the electrical performance of silicide contacts in doped silicon nanostructures. In particular, we compare these interfaces to ideal low-resistivity NiSi contacts of equivalent dimensions to evaluate their potential for nanoelectronic device integration. For the smallest nanostructures considered here, the total electrical contact resistance (**R**_**total**_) is primarily governed by the interfacial contact resistance (**R**_**C**_) which strongly depends on the interfacial surface area. According to eq (1) (see Discussion 5 in the Supporting Information), this implies that the increased surface area of faceted NiSi_2_ interfaces may lead to a lower **R**_**total**_ than that of flat NiSi contacts, despite the higher intrinsic resistivity of NiSi_2_.

The interfacial surface area was estimated through 3D reconstruction of the faceted interfaces observed in nanowires and nanosheets (background illustrations in Figure 5b,c) based on high-resolution rotational STEM analysis (Figure 5a). In the micrograph, two arrowhead-shaped features exhibit twinned facets upon sample rotation, indicating that they represent opposing faces of a single elongated pyramidal silicide domain. Based on systematic observations, nanowires were classified into three interface regimes for estimating the total interfacial surface: single facet (d_NW_ < 30 nm), single pyramid (d_NW_ 30–50 nm), and multipyramidals (d_NW_ > 50 nm).

**Figure 5:**
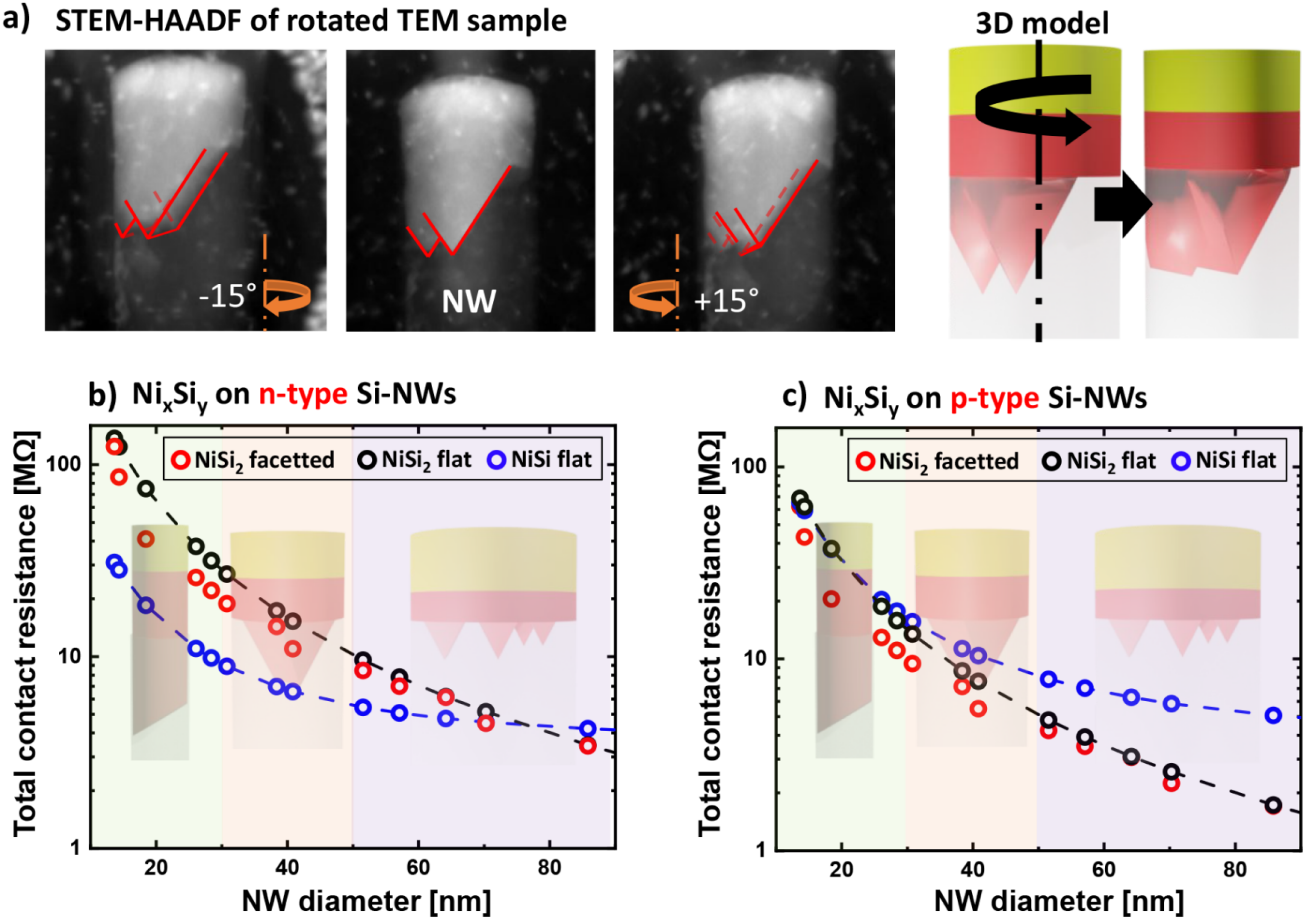
Estimation of the total electrical resistance of nanoscaled silicide contacts (*R*_total_) on *n*- and p-type vertical silicon nanowires, including silicide resistance and interfacial contact resistance R_C_. Rotational STEM-HAADF observations a) have been used to identify the elongated prism/pyramid geometry of faceted interfaces. In combination with the previous TEM observations, the interfacial surface area for simplified interface geometry models has been estimated to determine the *R*_total_ values on *n*- type NWs b) and p-type NWs c). These estimations are compared with theoretical values assuming either a flat NiSi_2_ interface or flat NiSi interface. Contact resistivity values for the estimation of NiSi and NiSi_2_ contacts are listed in Table S2.

Using these geometries, the total contact resistance was calculated for silicide contacts with mixed NiSi/NiSi_2_ compositions—representative of samples annealed at 400 °C—using eq (1) and literature-based resistivity (Table S1) and contact resistivity values (Table S2). These values were then compared to ideal planar contacts composed of either pure NiSi or the same NiSi/NiSi_2_ mixture (Figure 5b,c).

For both n-type and p-type silicon nanowires, the faceted NiSi_2_ interface offers a clear advantage over a flat NiSi_2_ interface, reducing **R**_**total**_ by approximately 30–45% for diameters in the 15–40 nm range. For larger nanowires, the influence of interface morphology becomes negligible, with reductions between 0 and 10%. When compared to a flat NiSi contact, the faceted NiSi_2_ interface yields notable performance gains in p-type silicon. Similar reductions are observed at small diameters, and even more significant reductions—up to 50–70%—are seen for larger diameters (55–90 nm). However, in n-type silicon, the inherently higher contact resistivity of NiSi_2_ leads to a 30–300% increase in total resistance for nanowires with diameters below 60 nm, compared to flat NiSi contacts.

Nevertheless, it remains an open question whether low-resistivity NiSi_2_ can be stabilized on n-type nanostructures, similar to epitaxial NiSi_2_ layers reported on p-type silicon. For instance, first-principles calculations predict that hyper-doped n-type silicon could achieve contact resistivities as low as 4 × 10^– 1 1^ Ω·cm^2^.^40^ If such low-resistance NiSi_2_ contacts can be realized, improvements in contact resistance of up to 100% could be expected across all nanowire diameters. Additional benefits might arise from geometric electron channeling through the sharp (001) pyramidal NiSi_2_/Si interface, as well as from crystallographic enhancements in electronic conductivity within the NiSi_2_ phase itself.^41^ Both effects could contribute to a further reduction in contact resistance, particularly in n-type silicon devices.

## Conclusion

This work presents, for the first time, a comprehensive understanding of how nanoscale confinement in vertical silicon nanostructures influences Ni-silicidation and alloy contact formation. By precisely depositing equivalent Ni volumes on nanostructures of varying sizes, we enabled a direct, comparative study of silicide formation across different geometries—overcoming limitations in earlier studies related to poor reproducibility and low comparability. Our results show that Ni-silicidation in confined vertical Si(001) nanostructures follows a distinct reaction path compared to planar substrates, with NiSi_2_ forming preferentially along the (111) planes even at temperatures as low as 300 °C. This behavior is driven by interfacial stress minimization, due to the low lattice mismatch between NiSi_2_ and Si, and by diffusion asymmetry due to fast Ni surface diffusion from a limited reservoir and self-limited Si diffusion through the growing NiSi_2_ layer. The dimensions of the nanostructures critically affect phase formation. Structures below a critical size—approximately 80 nm for nanosheets and more than 100 nm for nanowires—enhance Ni surface diffusion, promoting faceted NiSi_2_ growth and slowing Si counter-diffusion. This leads to a short-term stabilization of Ni-rich phases such as NiSi, Ni_2_Si, and potentially Ni_3_Si, which eventually convert to NiSi_2_ at higher temperatures or longer annealing durations. Additionally, the faceted NiSi_2_ interfaces improve electrical contact performance. On p-type Si, epitaxial (111) NiSi_2_ facets may reduce total contact resistance by up to 50–85%. For n-type Si, similar improvements may be possible in the case of electron channeling along (100) NiSi_2_ pyramid tips, particularly in nanowires with diameters of 30–50 nm, or through hyper-doping. These findings offer new insights into confined silicide growth and provide practical guidelines for optimizing contact interfaces in advanced 3D nanoscale CMOS technologies.

## Methods

Different vertical nanostructures including nanowires (NW) and nanosheets (NS) of different dimensions are fabricated by a top-down approach on unintentionally boron-doped (3 × 10^15^ cm^–3^) silicon (001) substrates. The patterns are defined by e-beam lithography (EBL) using a high-resolution negative-tone resist, namely Hydrogen Silsesquioxane (HSQ), in a Raith-150 system at minimal spot size and an acceleration voltage of 30 kV. After development in 25% Tetramethylammonium hydroxide (TMAH) and rinse in methanol, resulting structures are highly vertical. These nanopatterns are then transferred onto the substrate by an inductively coupled plasma etching (ICP-RIE) in an Alcatel AMS4200 system. The fluorine-based reactive ion etching (RIE) is perfectly anisotropic and used to create structures of 200–220 nm height. Any remaining HSQ is removed after submersion in diluted hydrofluoric acid, followed by a low-power oxygen plasma cleaning to remove any carbon residues introduced in the primary plasma etching process. Surface damages have been removed through the creation of a sacrificial oxide layer and its successive removal in a buffered oxide etchant (BOE) solution, also reducing the nanostructures’ dimensions. The sacrificial oxide is grown by dry-thermal oxidation in a Centronic E1550HT tube furnace, measuring 2.4 nm on bulk Si-substrate. Before the metal deposition, any native oxide on the prepared nanostructures is removed by dipping in diluted hydrofluoric acid followed by an in situ 2 min argon milling step within the deposition chamber of a Plassys MEB550SL PVD system. Nominally 10 nm of nickel are then deposited at a low deposition rate of 0.1 nm/s and a pressure of 6.5·10^–5^ mbar. The deposition occurs at room temperature without any additional heating and with a beam power loading of 280 mA and 10 kV. The silicidation process is activated by rapid thermal annealing (RTA) in an AS-One rapid-thermal processing furnace. Samples are heated up to 300–500 °C for 2 min under forming gas (3% H_2_/N_2_) atmosphere. The nanostructures are observed during the different process steps by scanning electron microscopy (SEM) in a FEI Helios 600i and finally characterized in detail after silicidation by transmission electron microscopy (TEM) in cross section. TEM investigations were performed by scanning TEM (STEM) in High Annular Dark-Field (HAADF) mode and by energy dispersive X-ray spectroscopy (EDX) in a JEOL JEM-ARM200F microscope as well as by 4D STEM in a JEOL JEM-2100F. The used TEM lamellas are prepared using a focused ion beam (FIB) in a FEI Helios 600i double beam system. A carbon-contrast layer of 50 nm is deposited on the structures followed by a protective Pt layer of more than 500 nm (see Figure S14).

## Supplementary Material



## Notes

Discussion 1: literature review on planar nickel silicides; Figure S1: schematic illustration of planar Ni-silicidation; Discussion 2: electrical measurements on planar Ni–Si thin film; Table S1: reference Ni–Si phase data; Figure S2: electrical measurements on planar Ni–Si thin film; Discussion 3: EDX reference measurements for signal intensity identification; Figure S3: EDX of planar Ni–Si; Figure S4: EDX and quantification of NiSi in planar silicide; Figure S5: EDX and quantification of Ni_2_Si in planar silicide; Figure S6: EDX and quantification of NiSi_2_ in nanostructured silicide; Figure S7: Phase conversion table for EDX intensities; Discussion 4: Identification of Ni–Si phases and NiSi_2_ facets by FFT; Figure S8: FFT and EDX characterization of as-deposited Ni thin films; Figure S9: FFT analysis of Ni_2_Si in planar silicide; Figure S10: FFT analysis of NiSi on large NS and planar NiSi; Figure S11: FFT analysis of NiSi_2_ facets at 500 °C; Figure S12: ACOM correlation results and raw maps for Ni–Si phases; Figure S13: Extended Ni–Si reaction process illustration; Discussion 5: Contact resistance equation and literature resistivity values; Table S2: Literature reference values for Ni–Si contact resistivities; Figure S14: Nanostructured TEM lamella preparation (PDF)

## #

Deceased 12.01.2024.
