# Supplementary material for: Engineering Ni-Silicide Nanocontacts for 3D Silicon Devices via Geometrical Confinement Control
Source: ACS Nano. 2025 Jul 31;19(31):28450–9. doi: 10.1021/acsnano.5c07195 (PMC12356115; doi:10.1021/acsnano.5c07195)
Supplement: Supplementary file 1 [file nn5c07195_si_001.pdf]

Supporting information:

Engineering Ni-Silicide Nanocontacts for 3D Silicon Devices via Geometrical Confinement Control

Jonas Müller[1], Remi Demoulin[1], Leonardo Cancellara[1], Fuccio Cristiano[1,†], and Guilhem Larrieu[1,*]

[1] LAAS-CNRS, University of Toulouse, CNRS, Toulouse 31031, France

* Corresponding author; Email: guilhem.larrieu@laas.fr

Table of content:

Supporting information: 1
Engineering Ni-Silicide Nanocontacts for 3D Silicon Devices via Geometrical Confinement Control 1
Table of content: 1
Supporting Information Discussion 1. Literature review on planar nickel silicides 2
Supporting Information Figure S1. Schematic illustration of planar Ni-silicidation 3
Supporting information Discussion 2. Electrical measurements on planar Ni-Si thin film 3
Supporting information Table S1. Reference Ni-Si phase data 5
Supporting information Figure S2. Electrical measurements on planar Ni-Si thin film 6
Supporting information Discussion 3. EDX reference measurements for signal intensity identification. 6
Supporting information Figure S3. EDX of planar Ni-Si 8
Supporting information Figure S4. EDX & quantification of NiSi in planar silicide 9
Supporting information Figure S5. EDX & quantification of Ni_2Si in planar silicide 9
Supporting information Figure S6. EDX & quantification of $NiSi_2$ in nanostructured silicide 10
Supporting information Figure S7. Phase conversion table for EDX intensities 10
Supporting information Discussion 4. Identification of Ni-Si phases and $NiSi_2$ facets by FFT 10
Supporting information Figure S8. FFT and EDX characterization of as-deposited Ni thin films 12
Supporting information Figure S9. FFT analysis of Ni_2Si in planar silicide 12
Supporting information Figure S10. FFT analysis of NiSi on large NS and planar NiSi 13
Supporting information Figure S11. FFT analysis of $NiSi_2$ facets at 500°C 13
Supporting information Figure S12. ACOM correlation results & raw maps for Ni-Si phases 14
Supporting information Figure S13. Extended Ni-Si reaction process illustration 14
Supporting information Discussion 5. Contact resistance equation & literature resistivity values. 15
Supporting information Table S2. Literature reference values for Ni-Si contact resistivities 16
Supporting information Figure S14. Nanostructured TEM lamella preparation 16
References 17

† Deceased 12.01.2024

Supporting Information Discussion 1. Literature review on planar nickel silicides

The conventional Ni-Si transformation sequence as described in the literature is as follows: During the silicidation reaction, Ni is the initial diffusing component that enters the substrate. Thereby firstly forming Ni-rich phases such as Ni_3Si and Ni_2Si, depending on the used annealing method. The mixing process is controlled by the interstitial diffusion of nickel atoms into the silicon substrate and a successive complex dissociative diffusion mechanism that introduces nickel into substitutional position through defect or vacancy interactions for a sufficient thermal activation [1, 2]. The initial interstitial diffusion occurs already at room temperature [3] due nickel's high room-temperature diffusivity [4].

For the conventional process nickel-rich Ni_2Si forms first at 200-350 °C [5] and transforms first in to NiSi at 400 °C and for temperature past 550 °C in to $NiSi_2$ [6, 7, 8]. NiSi appears as the dominant phase for processing temperatures between 350-550 °C [8] but can also be observed for higher reaction temperatures, depending on the annealing duration [6, 9, 10]. In industrial processes, NiSi silicidation processes are carried out at an annealing temperature of around 500 °C for a few minutes to fully transform the nickel into NiSi, based on the transformation sequence indicated in Figure S1a. The conventional Ni-silicidation sequence is however largely simplified, neglecting the nickel-rich phases such as Ni_3Si, $Ni_{31}Si_{12}$ and Ni_3Si_2 which may appear during rapid annealing processes (RTP or RTA). Using low temperature RTP, intermediate phases such as the high resistive $Ni_{31}Si_{12}$ and Ni_3Si_2 have been observed by x-ray diffraction [11] whereas Ni_3Si has recently been found to already form at room temperature by simulation and X-ray reflectometry [12]. Consequently, complex/partial transformations may occur such as the simultaneous decomposition of $Ni_{31}Si_{12}$ into Ni_2Si and Ni_3Si_2 in order to reduce the mismatch stress in the system [13]. A schematic Ni-silicide phase transformation sequence for the rapid thermal annealing process is presented in Figure S1b.

The formation of pyramidal silicide as presented in the main text (and in similar works on nanostructures [14, 15, 16, 17]) has also been observed for the Ni supply-limited growth in thin films [18], including Ni-Si reactions through a diffusion barrier reported in [19, 20] or for ultra-thin layers [21]. Identical $NiSi_2$ growth with facets along the [111] direction of the Si substrate was observed and appeared at significantly lower formation temperatures as compared to 800°C in planar samples. This shift is a consequence of higher dimensional constraints in the ultrathin layers which can lower the $NiSi_2$ formation temperature down to 300°C for ultra-thin Ni layer below 2.5 nm thickness [21] or 300-500°C for diffusion barriers at the interface [19].

Supporting Information Figure S1. Schematic illustration of planar Ni-silicidation

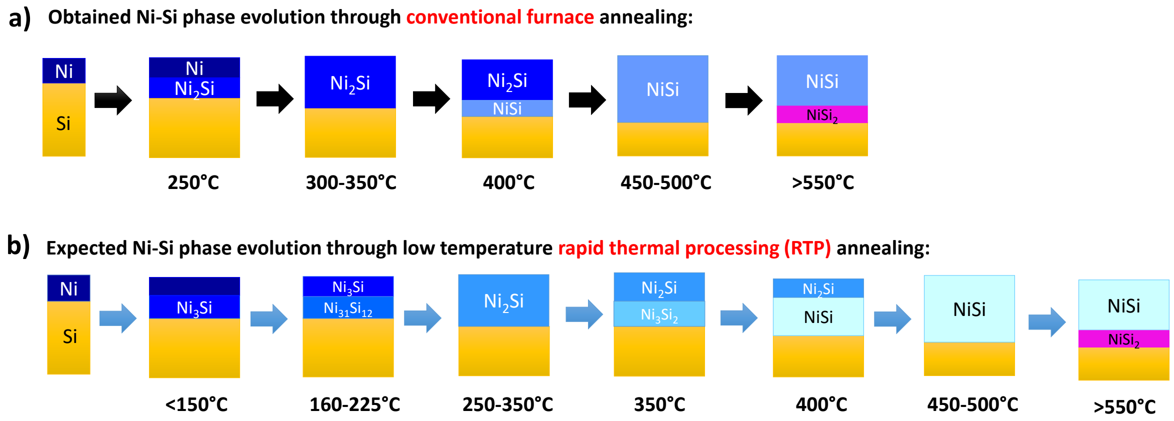

Figure S1. *Schematic representation of the traditionally observed phase transformation sequence using conventional furnace annealing (a) compared to rapid thermal processing (RTP) (b) of bulk Ni-silicide. Faster annealing processes expectedly give rise to the appearance of more Ni-rich and intermediate phases. The indicated temperatures represent the temperature ranges for new silicide phases to emerge. Exact transition temperatures vary depending on multiple factors e.g. the chosen annealing time as well as the substrate (doping, defects, etc.).*

Supporting information Discussion 2. Electrical measurements on planar Ni-Si thin film

Silicide thin film sample fabrication: Full wafers of lightly Boron doped Silicon wafers (4" (100) p-Si, Boron concentration 1-2·10^{19} cm^{-3}) are firstly cleaned in Piranha (H_2SO_4:H_2O_2 1:1) solution to remove any organic residues on the surface. Secondly, the native silicon oxide layer of 1-2 nm thickness is removed by wet chemical etching in diluted Hydrofluoric acid

(HF(5%):Methanol 1:1) for 30s. Prepared wafers are immediately placed under vacuum in an electron-beam evaporator “Plassys MEB-550SL” to minimize the re-formation of the native oxide layer. An in-situ Ar milling (5 sccm flow of Ar with a chamber pressure of $6.5 \cdot 10^{-5}$ mbar for 2 min) is performed to remove any oxide traces prior the metal deposition. A 10 nm thick nickel thin film is afterwards deposited as described in the “Methods” section and the obtained wafer is cut into $2 \cdot 2$ cm^2 chips. Individual chips are then submitted to RTA at temperatures of 200-800 °C for 0.5-10 minutes.

Electrical characterization of planar Ni-Si: The electrical properties of the formed silicide layers were characterized by sheet resistance measurements using a CMT-SR2000NW four-point probe station. The results, covering annealing temperatures from 200 °C to 800 °C and annealing times of 60 and 120 seconds, are presented in Figure S2. These measurements are compared to literature data summarized in Table S1 and show good agreement with previous studies on silicide formation under comparable RTA conditions [6, 22]. At low annealing temperatures, the sheet resistance initially exhibits a slow increase, reaching a maximum around 300 °C. This behavior is attributed to the partial transformation of Ni-rich silicides, such as Ni_3Si and $Ni_{31}Si_{12}$, into more stable phases. A subsequent sharp decrease in resistance indicates the formation of Ni_2Si, followed by the emergence of a low-resistance plateau beginning around 350–400 °C, corresponding to the formation of the NiSi monosilicide phase. The onset temperature of NiSi formation is annealing-time dependent: for prolonged durations (~10 minutes), this transition occurs at temperatures as low as 300 °C, as illustrated in the inset of Figure S2. The NiSi phase remains stable up to approximately 600 °C, beyond which the sheet resistance increases sharply. This rise is attributed to morphological degradation and agglomeration of the NiSi layer, which may also lead to transformation into $NiSi_2$ at elevated temperatures. Reference studies [6, 22] report higher thermal stability of the

NiSi phase, extending up to 800 °C. These discrepancies may arise from differences in annealing conditions, such as the ramp rate, ambient atmosphere, or substrate doping, or from intrinsic thin-film effects (e.g., stress or interfacial energy) that promote earlier degradation and phase transformation in our samples. For example, Reference [6] used 15 nm Ni on polycrystalline Si annealed at 3 °C/s under a purified He atmosphere, while Reference [22] employed 10 nm Ni on either undoped or B-implanted Si, annealed under N_2 for 60 seconds.

Supporting information Table S1. Reference Ni-Si phase data

Table S1. *List of the seven out of eleven stable Ni-Si phases to be formed after low-temperature annealing processes [6, 9] with relevant data for material/phase characterization. Resistivity measurements are taken from [7, 23, 24, 25, 26, 27, 28, 29, 30, 31] and crystallographic data for FFT analysis is taken from MaterialsProject [32] and SpringerMaterials [33]. The solubility of Ni in silicon substitutional lattice sites is taken from [34] as determined at 1000°C.*

Phase	Density [g/cm^3]	ρ [μΩ·cm]	Silicide ratio [t/t]	Si cons. [t/t]	Ni content [at. %]	Crystal structure	Space group	Lattice constants [Å]
Ni	8.91	7-10	1	0	100	Cubic	Fm3'm	2.48/ 2.48/ 2.48
Ni_3Si	7.78	80-90	1.31	0.61	75	Cubic	Pm3m	3.51/ 3.51/ 3.51
$Ni_{31}Si_{12}$	7.56	90-150	1.40	0.71	72.1	Hexag.	P321	6.65/ 6.65/ 12.28
Ni_2Si	7.51	24-30	1.47	0.91	58-67	Hexag.	$P6_3$/mmc	3.90/ 3.90/ 4.98
					66.7	Ortho.	Pnma	3.73/ 4.98/ 7.07
Ni_3Si_2	6.71	60-70	1.75	1.22	59-61	Ortho.	Cmc21	10.70/ 12.10/ 6.87
NiSi	5.97	10.5-18	2.20	1.83	50	Ortho.	Pnma	3.35/ 5.16/ 5.58
$NiSi_2$	4.80	34-50	3.61	3.66	33.3	Cubic	Fm-3m	5.41/ 5.41/ 5.41
Si	2.33	$6.4 \cdot 10^6$	-	-	$< 2 \cdot 10^{-3}$	Cubic	Fd-3m	5.43/ 5.43/ 5.43

Supporting information Figure S2. Electrical measurements on planar Ni-Si thin film

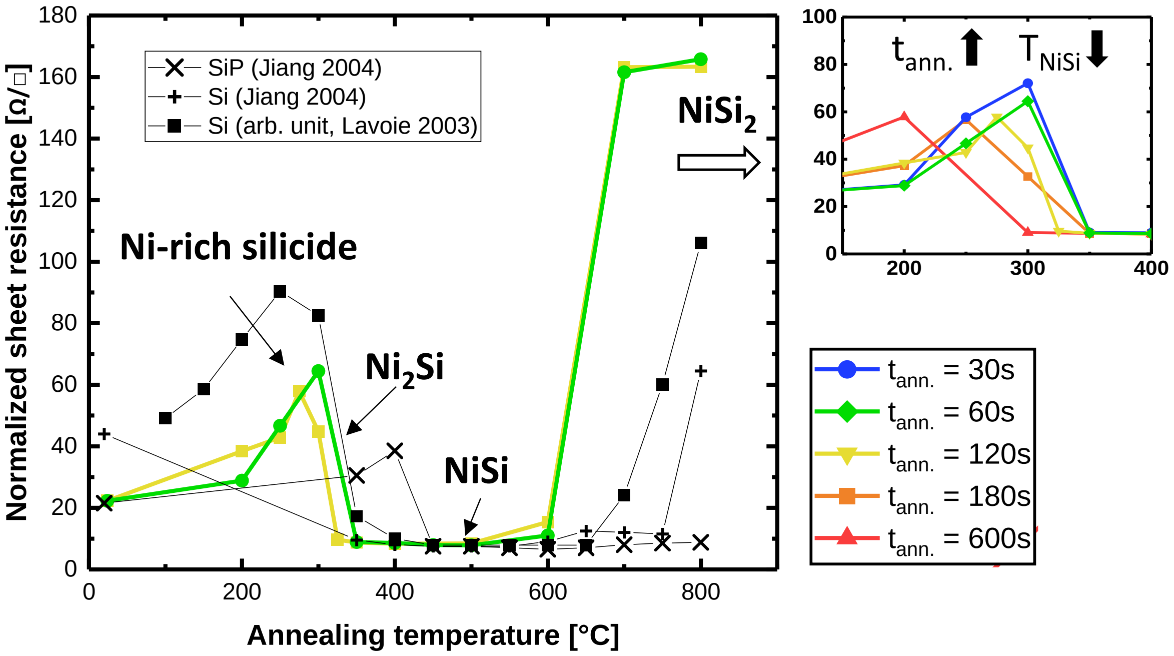

Figure S2. *Sheet resistance measurements of bulk silicide reference samples as a function of rapid thermal annealing (RTA) temperature and annealing time (t_{ann}). The dominant silicide phases (Ni_2Si, NiSi) were identified based on their known resistivities (see Table S1). The results exhibit the expected sequence of Ni-Si phase transformations with increasing temperature and are in good agreement with literature data, including those reported by Jiang et al. [22] and Lavoie et al. [6]. For extended annealing durations, the onset of the transition from Ni-rich silicides to NiSi is observed to shift toward lower temperatures, as highlighted in the magnified inset (right panel).*

Supporting information Discussion 3. EDX reference measurements for signal intensity identification.

The planar Ni-Si silicide layers surrounding the nanostructures of each annealed nanostructure sample as well as a Ni reference thin film have been characterized by 4-Probe measurement and STEM analysis (Figure S3a). The sheet resistance of the Ni-thin film after deposition is 22.6 Ω/□. The sheet resistances of the formed silicide layers are measured as 14.2, 10.6 and 10.6 Ω/□ after annealing at 300, 400 and 500 °C respectively. Based on those measurements a full

NiSi transformation after RTA at 400/500 °C is expected while at 300 °C Ni_2Si should still be present. The thicknesses of the silicide layers have been measured as 10.6 for Ni as-deposited and 21.4, 21.8 and 25.5 nm respectively. All silicide layers show an interfacial diffusion layer Si(Ni) of about 4-5 nm.

The planar Ni-Si silicide composition of the three annealed samples has been analyzed by STEM-EDX, measuring the raw intensities profiles of Ni, Si and O as shown in Figure S3b. The EDX profiles indicate a homogenous Ni:Si ratio at 400 and 500°C corresponding to NiSi and an increased Ni-content for the sample annealed at 300°C. All samples exhibit the presence of both silicon and oxygen at the surface, consistent with the formation of a native silicon oxide layer atop the silicide. This oxidation occurs after the silicidation annealing step, during sample handling and ambient exposure prior to focused ion beam (FIB) preparation for TEM analysis. It is well established that oxygen can disrupt surface Ni–Si bonds, promoting the formation of a metal-free silicon oxide layer while enabling the released Ni atoms to diffuse into the underlying silicon substrate. [35, 36, 37, 38].

The raw EDX signals of the planar silicides of Figure S3b were used to calculate the Ni:Si intensity ratios in Figure S4a and to quantify the Ni:Si composition in Figure S4b. The main phase of all samples is NiSi: For 400 °C, the NiSi layer is uniform and measures 21.8 ± 0.4 nm. A similar overall thickness of 21.4 ± 0.4 nm is found at 300 °C but the quantified EDX reveals a partially transformed Ni-rich layer of ca 8.6 nm near the surface. TEM images support the presence of granular Ni_2Si embedded in NiSi rather than a homogeneous Ni_2Si layer. At 500 °C, the silicide surface has a decreased Ni concentration which may be correlated with silicide agglomeration or "void" formations as well as an interfacial layer in between the silicon substrate, yielding an enhanced thickness of 25.5 ± 0.6 nm. At the NiSi/Si interface, regardless

of RTA temperature, a smooth Ni-gradient indicates a Ni-diffusion/mixing layer formation of low Ni content (< 10% Ni).

Using EDX results presented in Figure S4-6, a classification table for the conversion of raw intensity signal ratios and quantified compositions has been created. This table is used to determine the phase composition in small structures for which only raw intensity EDX line scans are available as a software-based quantification is not possible, due to the low signal to noise ratios. Intensity values for NiSi are taken from planar silicide regions of nanostructured samples (Figure S4), values for Ni_2Si from planar silicide reference samples (Figure S5) and $NiSi_2$ from planar-like nanosheet (not shown) and nanowire samples (Figure S6). The final results are summarized in Figure S7a. The additional intensity ratio for Ni-rich phases (Ni_3Si and $Ni_{31}Si_{12}$) have been estimated by fitting the intensity ratio over the equivalent atomic number of the silicide phases as taken from [39] (see Figure S7b). Due to the resulting uncertainty for the intensity identification of Ni_3Si and $Ni_{31}Si_{12}$, both are referred to as $Ni_{(x>2)}Si$.

Supporting information Figure S3. EDX of planar Ni-Si

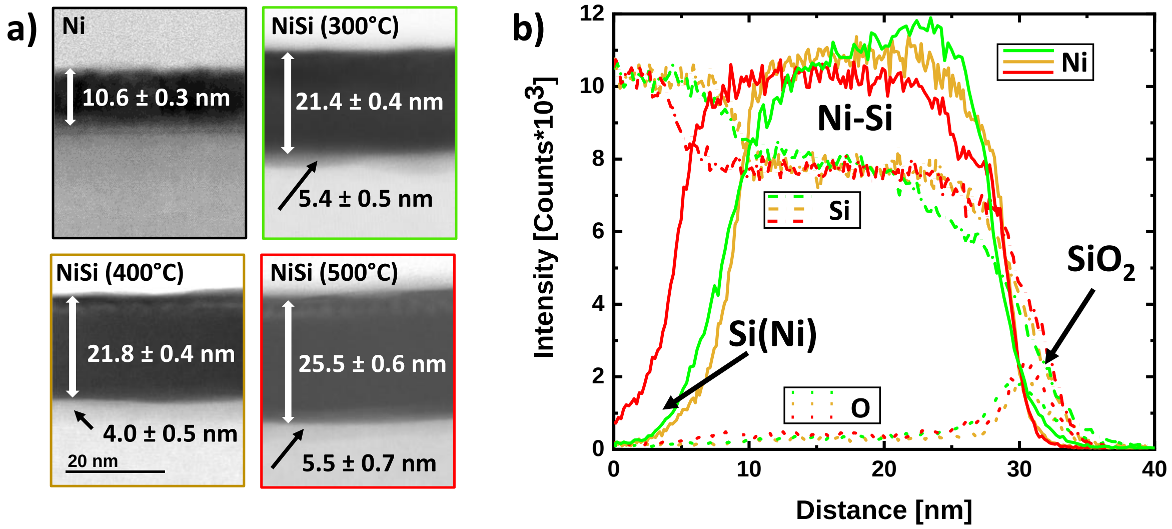

Figure S3. *STEM bright-field images of the planar Ni as deposited and Ni-Si silicide layers after annealing (a). The three annealed silicide samples have been characterized by EDX as presented by the averaged EDX intensity signals in (b).*

Supporting information Figure S4. EDX & quantification of NiSi in planar silicide

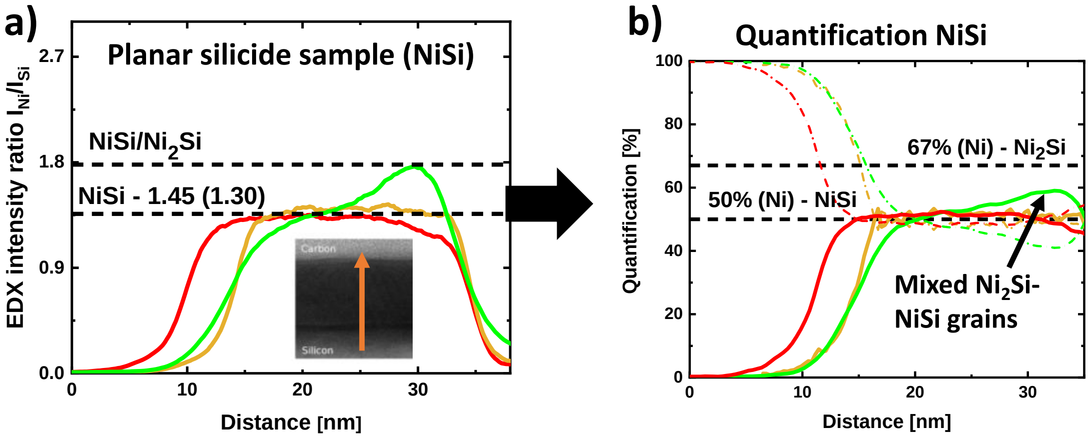

Figure S4. *EDX analysis of Ni-Si on planar reference samples. The Ni-Si composition obtained after different annealing processes is presented by the EDX intensity ration I_{Ni}/I_{Si} for planar and NS samples in a). All reference samples were quantified as shown in b) confirming the EDX intensity ratios of the corresponding Ni-Si phases. The visible EDX intensity peak of Ni_2Si stems from Ni_2Si grains embedded in NiSi near the surface which yields a decreased average Ni concentration of 60 at% by quantification. Used reference values for intensity identification Max(Min) of NiSi are noted in a).*

Supporting information Figure S5. EDX & quantification of Ni_2Si in planar silicide

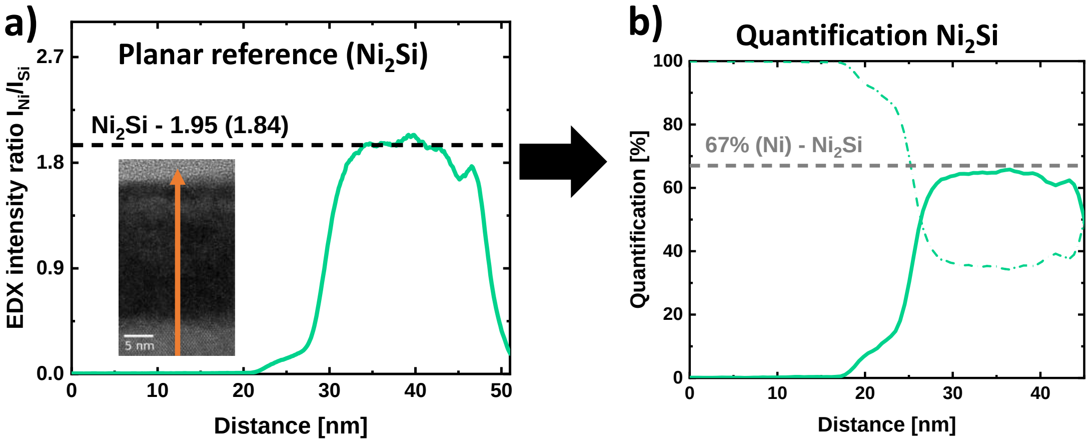

Figure S5. *EDX analysis of a planar silicide reference for Ni_2Si. The corresponding plots for the EDX intensity ration I_{Ni}/I_{Si} and the quantified composition are shown in a) and b) respectively. Used reference values for intensity identification Max(Min) of Ni_2Si are noted in a).*

Supporting information Figure S6. EDX & quantification of $NiSi_2$ in nanostructured silicide

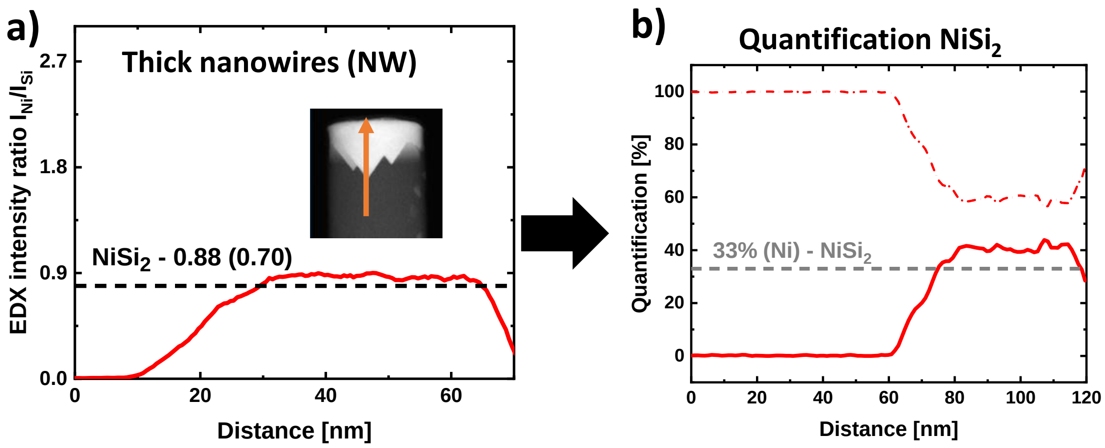

Figure S6. *EDX analysis of fully transformed $NiSi_2$ in large silicon nanowires (d_{NW} > 100 nm), where the nanowire width exceeds the thickness of the TEM lamella. (a) Ni:Si EDX intensity ratio (I_{Ni}/I_{Si}) and (b) the corresponding quantified atomic composition. Reference threshold values used for phase identification—denoted as Max(Min) for $NiSi_2$—are indicated in (a).*

Supporting information Figure S7. Phase conversion table for EDX intensities

a) Measured & estimated EDX intensity ratios:

Phase	Composition (Ni/Si) [at%]	Z^2N [$10^{25}/cm^3$]	Measured I_{Ni}/I_{Si}	Target I_{Ni}/I_{Si}
Ni_3Si	75/25	5.70	-	2.23 ± 0.05
$Ni_{31}Si_{12}$	72/28	5.28	-	2.04 ± 0.05
Ni_2Si	67/33	4.99	1.84-1.95	1.90 ± 0.08
Ni_3Si_2	60/40	-	-	-
NiSi	50/50	3.66	1.30-1.45	1.38 ± 0.11
$NiSi_2$	40/60	2.66	0.70-0.88	0.79 ± 0.13

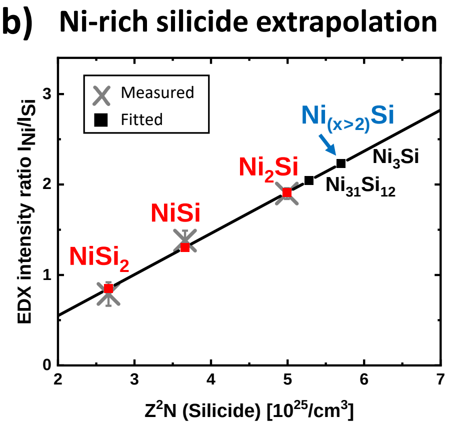

Figure S7. *(a) Reference table for the identification of Ni–Si phases based on estimated Ni:Si EDX intensity ratios (I_{Ni}/I_{Si}). The measured intensity ranges were obtained from various reference samples and nanostructures, as shown in Figures S4–S6. Experimental target values for Ni_2Si, NiSi, and $NiSi_2$ (highlighted in red) were determined from directly measured I_{Ni}/I_{Si} ratios. In contrast, the target intensity ratio for Ni-rich phases was extrapolated, as illustrated in (b), based on the silicide's equivalent atomic number Z^2N [39]. The extrapolated ratio for Ni_3Si is used as an indicative reference for the identification of Ni-rich silicides, denoted as $Ni_{(x>2)}Si$ and highlighted in blue.*

Supporting information Discussion 4. Identification of Ni-Si phases and $NiSi_2$ facets by FFT

Additional verification of the crystallinity and phase identification of the Ni–Si compounds, namely, the deposited Ni thin films (Figure S8), planar Ni_2Si (Figure S9), NiSi on planar Si and

vertical nanosheets (Figure S10), and $NiSi_2$ in large vertical nanowires (Figure S11), was performed using fast Fourier transform (FFT) analysis of high-resolution (S)TEM micrographs. The resulting diffraction patterns were indexed using the "diffractGUI" module of the CrysTBox software suite [40]. This approach enabled robust structural identification in structures with sufficient silicide volume. However, similar characterization could not be applied to smaller nanostructures, where the limited silicide thickness prevented acquisition of high-quality diffraction patterns. In contrast, for large nanowires with diameters exceeding the TEM lamella thickness and annealed at 500 °C, it was possible to resolve crystalline diffraction features from well-formed silicide facets. As shown in Figure S11, the bright-field high-resolution STEM image of the thickest nanowires revealed clear diffraction spots consistent with the $NiSi_2$ phase. In this case, the silicide prism likely extends across the full lamella thickness, thereby providing a sufficient volume for reliable structural characterization. The observed 110 plane of the Si crystal and the facet are compared and show a similar diffraction pattern. This is expected as Si and $NiSi_2$ have the same crystal structure with only a minor difference in the lattice parameters for $NiSi_2$. It is however clearly visible that the Si crystal has weak/absent reflections at the (002) positions which are forbidden reflections in silicon crystals [41]. In real crystals, these reflections may appear for silicon due to added scattering of defects or other scattering sources. As in Figure S9b,c for FFTs obtained from high-resolution STEM images, the weak/forbidden reflections are absent in the bulk silicon, but the reflections appear in the facet as a consequence of the additional scattering at Ni atoms. Our results match the HR-TEM and FFT results for planar $NiSi_2$ thin films formed at 510-575°C presented in [42]. In conclusion, FFT images obtained from HR-STEM images provide further proof, in addition to the chemical composition obtained by EDX, that the facets formed after annealing at 500°C are $NiSi_2$.

Supporting information Figure S8. FFT and EDX characterization of as-deposited Ni thin films

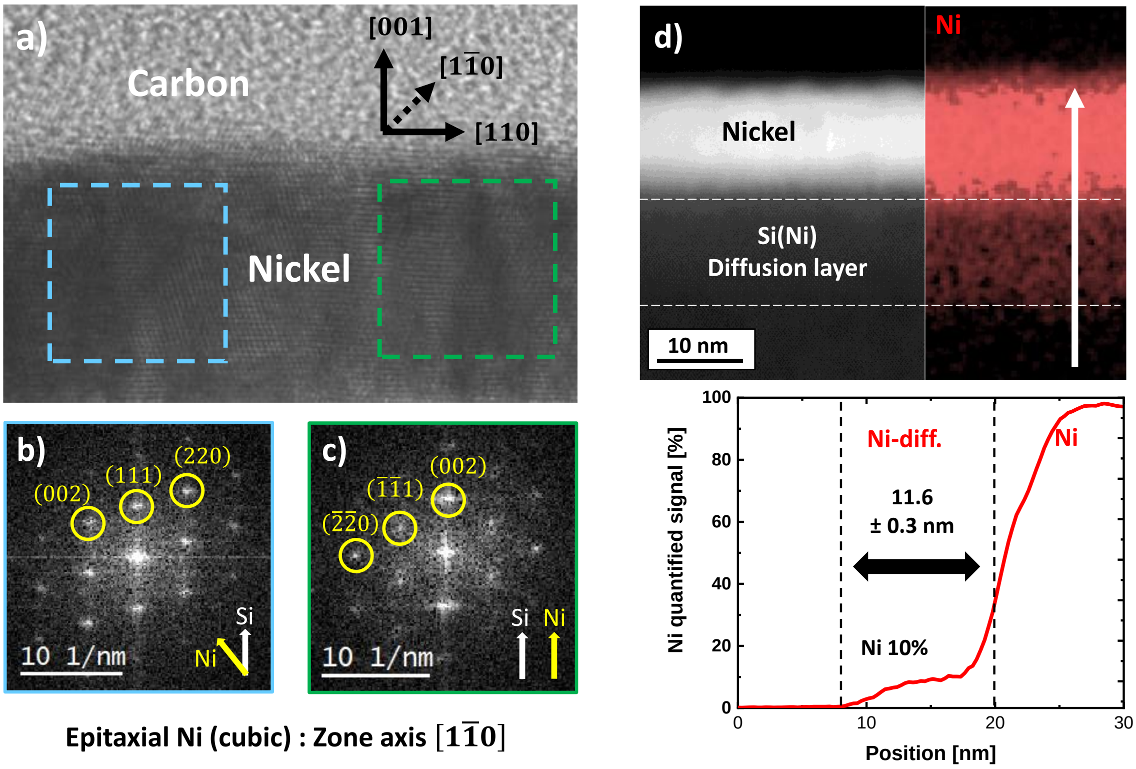

Figure S8. *Complimentary TEM and FFT characterization of the as-deposited Ni on bulk silicon substrate. The cubic crystal phase of nickel layer and silicon substrate have been verified by FFT in b, c) and d) respectively. The Ni grains of 10-15 nm width are epitaxially aligned with the substrate while few grains have varying orientations. d) STEM-HAADF observation and EDX map of the as-deposited Ni on bulk silicon substrate. Nickel has diffused into the Si substrate and can be detected 11.6 ± 0.3 nm below the Ni-Si interface with an atomic concentration of 10%. The Ni-atoms are assumed to be within interstitial Si-lattice sites.*

Supporting information Figure S9. FFT analysis of Ni_2Si in planar silicide

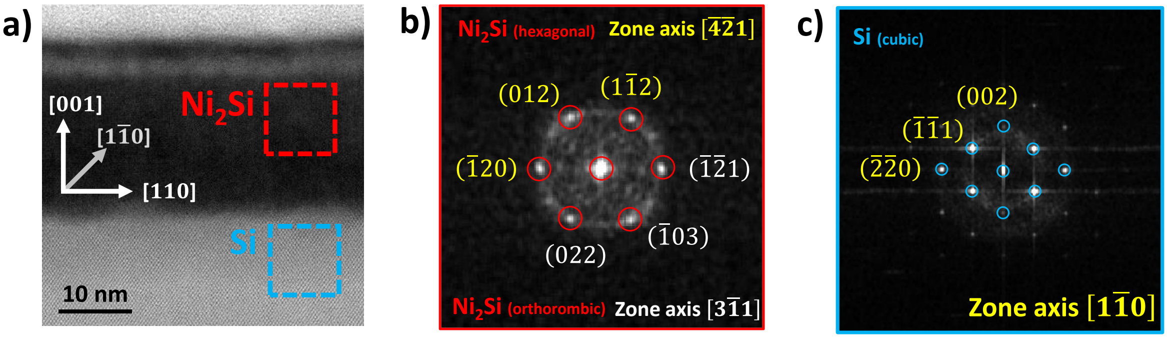

Figure S9. *Complementary TEM and FFT characterization of planar Ni_2Si formed on a bulk silicon substrate. The chemical composition of the silicide was verified by EDX, as shown in Figure S5. The silicide layer is polycrystalline, and multiple grains were identified as Ni_2Si. For the representative grain shown in (b), FFT analysis yielded a good match with both the hexagonal and orthorhombic Ni_2Si phases, consistent with known structural polymorphism.*

Supporting information Figure S10. FFT analysis of NiSi on large NS and planar NiSi

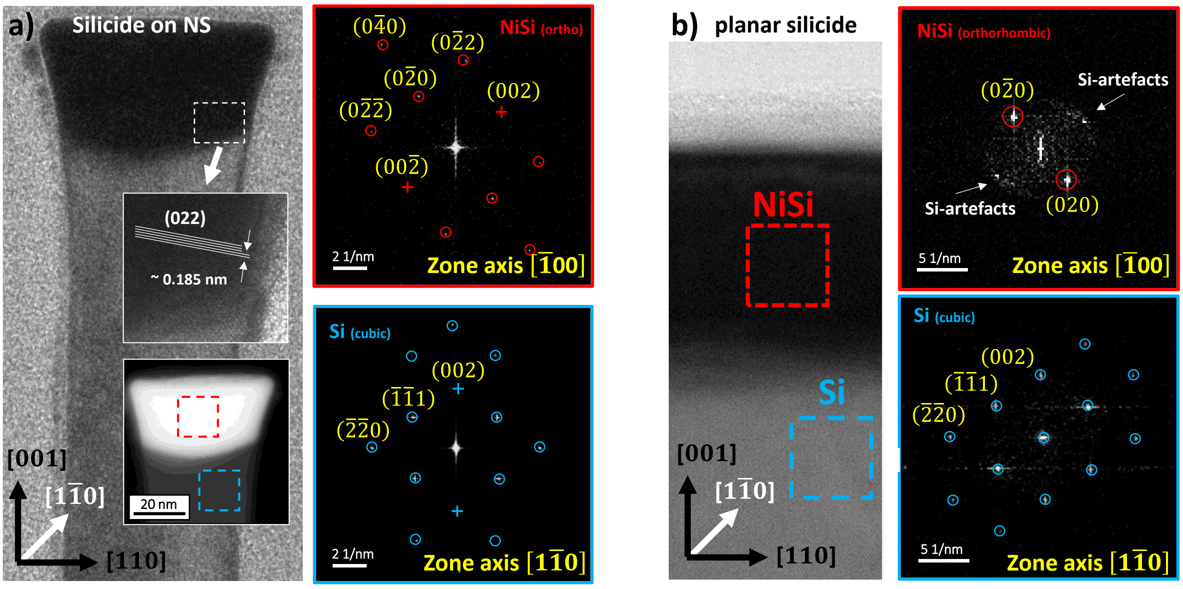

Figure S10. *Supplementary FFT analysis of large volume NiSi on vertical nanosheets (a) and planar silicon substrate (b) for silicide samples annealed at 400°C. The NiSi crystal structure in (a) is identified as orthorhombic and appears to be mono-crystalline. Diffuse diffraction rings in (b) indicate that the planar silicide is poly-crystalline. Two strong reflections, matching the (020) planes as seen for the NS FFT can be identified for certain grains, confirming the same crystal structure as observed in the NS.*

Supporting information Figure S11. FFT analysis of $NiSi_2$ facets at 500°C

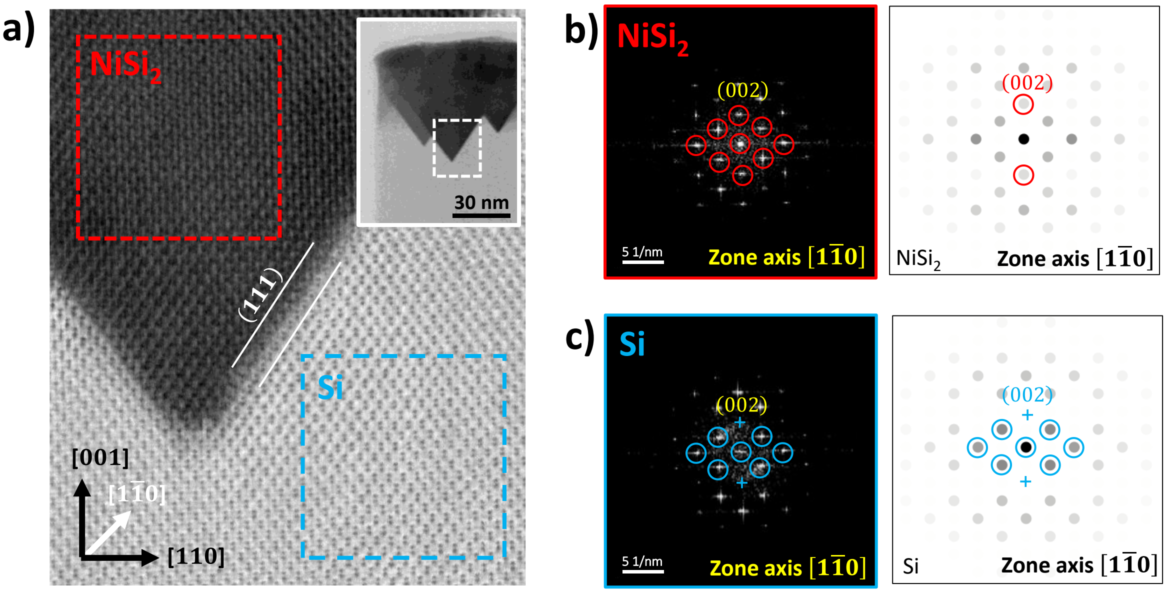

Figure S11. *FFT analysis of bright-field HR-STEM micrograph (a) for facetted Ni-Si alloy on vertical nanowires after RTA at 500°C. The recorded FFTs for the substrate (c) and the facetted phase (b) are identified as Si and $NiSi_2$ Si by comparison with calculated diffraction patterns retrieved from Materials project [43]. While the pure Si crystal shows weak (002) reflections, these are present for the $NiSi_2$ phase which has otherwise similar crystal structure to Si.*

Supporting information Figure S12. ACOM correlation results & raw maps for Ni-Si phases

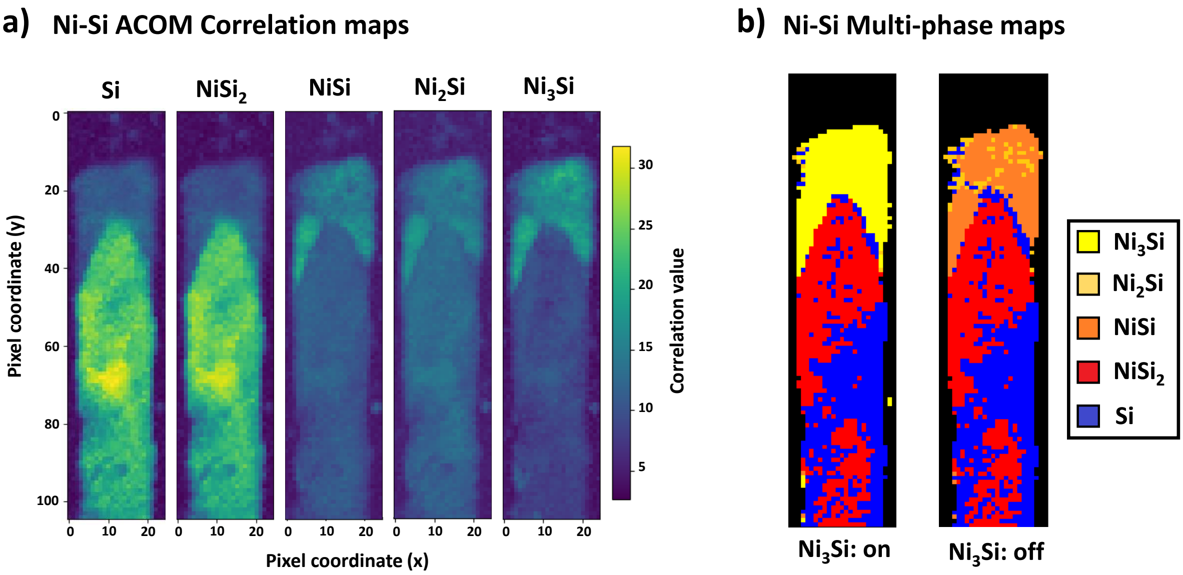

Figure S12. *Supplementary maps of the 4D-STEM characterization of the Ni-Si alloy presented in Figure 4a. (a) ACOM correlation maps results are displayed for all investigated phases (Ni_3Si, Ni_2Si, NiSi, $NiSi_2$, Si) on a highly confined vertical nanowire. Correlation results are similar for $NiSi_2$/Si as well as Ni-rich phases NiSi/Ni_2Si/Ni_3Si. Ni_3Si correlation values are artificially high due to an overlap of first order reflections of NiSi/Ni_2Si and Ni_3Si. (b) Unmodified multi-phase maps generated from all correlation maps ("Ni_3Si: on") thus falsely attribute Ni_3Si to all Ni-rich pixels. The Ni_3Si phase has thus been suppressed ("Ni_3Si: off") to generate the phase map as shown in Figure 4a.*

Supporting information Figure S13. Extended Ni-Si reaction process illustration

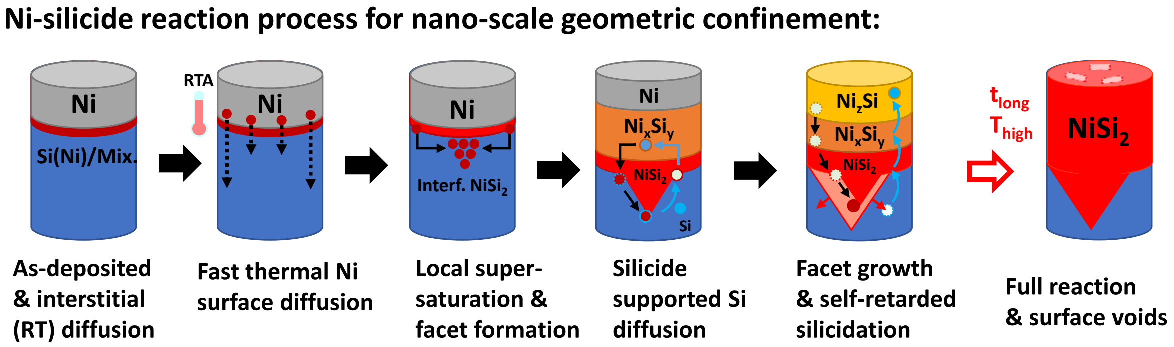

Figure S13. *Detailed Ni-Si reaction process on highly confined vertical nanostructures. Ni-atoms diffuse interstitially into the Si-substrate after deposition at room-temperature. The silicide formation is activated by rapid thermal annealing. The initial reaction is controlled by the fast Ni diffusion on the NS/NW surface and silicide interface. The supersaturation of Ni atoms at the interface then triggers an initial growth of $NiSi_2$ and the self-retarded Si-diffusion through the silicide. The extensive growth of the facetted $NiSi_2$ interface slows down the upward Si diffusion causing the appearance of Ni-rich phases in smallest nanostructures as observed after short RTA annealing times. It is expected that the alloy completely transforms into $NiSi_2$ for long annealing durations and/or higher temperatures as is the case for wider nanostructure dimensions.*

Supporting information Discussion 5. Contact resistance equation & literature resistivity values.

The contact resistance cited in the main text is expressed as follows:

$$\boldsymbol{R_{total}} = \boldsymbol{R_C} + \boldsymbol{R_{Silicide}} = \frac{\boldsymbol{\rho_c}}{\boldsymbol{S}} + \sum_{\boldsymbol{Ni_xSi_y}} \left(\frac{\boldsymbol{\rho}}{\boldsymbol{A}} \cdot \boldsymbol{t}\right) \qquad (1)$$

where $\boldsymbol{\rho_c}$ is the specific contact resistivity (to Si), ***S*** the surface of the silicide/silicon interface, ***A*** the cross-sections of the nanostructure, ***t*** the thickness of a certain silicide layer and $\boldsymbol{\rho}$ that silicide's resistivity.

Reported literature values for contact resistivities of nanometric thin films vary greatly as they depend on substrate doping, film thickness/constraints, annealing conditions and other supplementary fabrication parameters. Reported results for thicker nickel films of around 100 nm for example report high contact resistivities of the order of 10^{-3} $\Omega\cdot cm^{-2}$ for $NiSi_2$ [44] while $NiSi_2$ formed from thinner metal films leads to a decreased contact resistivity down to 10^{-7} $\Omega\cdot cm^{-2}$ [45]. In the case of ultrathin Ni-Si layers (using less than 5 nm of Ni) on highly doped SOI layers, $NiSi_2$ has been reported to achieve a similar contact resistivity as NiSi [46], which is true for p-type silicon. This is possible thanks to the formation of pure epitaxial $NiSi_2$ which also achieves a similar resistivity to NiSi due to its defect-free crystal structure [47]. Depending on the NW diameters, resistivities for $NiSi_2$ as low as 22.5 µΩ·cm (see Table S1 for comparison) have been demonstrated due to the reduced interfacial scattering [47]. Consequently, it is also to be expected that epitaxial $NiSi_2$ should exhibit a reduced contact resistivity close to NiSi. Representative experimental references for the contact resistivity of NiSi and $NiSi_2$ on highly doped p-/n-type substrates and for different Ni film thickness are listed in Table S2. For the

presented estimations in the main text, we rely on the measured contact resistivities taken from [45] representing a similar initial metal film thickness of 20 nm.

Supporting information Table S2. Literature reference values for Ni-Si contact resistivities

Table S2. *Literature values for the contact resistivity of NiSi and $NiSi_2$ on doped p-type and n-type silicon substrates, obtained for various silicide thicknesses. The initial Ni metal thickness prior to silicidation is also indicated. Values used in the manuscript's contact resistance estimation are highlighted.*

Phase	Contact resistivity ρ_c [$\Omega\cdot cm^2$]	Doping [cm^{-3}]	Ni thickness [nm]	Ref.
**NiSi**	**$4\cdot10^{-8}$**	**(n+) $2\cdot10^{20}$**	**20**	[45]
**NiSi**	**$9\cdot10^{-8}$**	**(p+) $1\cdot10^{20}$**	**20**	[45]
NiSi	10^{-5}-10^{-8}	(n+) 10^{19}-10^{20}	10	[48]
NiSi	10^{-5}-10^{-8}	(p+) 10^{19}-10^{20}	10	[48]
NiSi	$1\cdot10^{-5}$	(n+) > 10^{20}	5	[46]
NiSi	$5\cdot10^{-6}$	(p+) > 10^{20}	5	[46]
$NiSi_2$	$3.6\cdot10^{-3}$	(n+) < 10^{15}	100	[44]
**$NiSi_2$**	**$2\cdot10^{-7}$**	**(n+) $2\cdot10^{20}$**	**20**	[45]
**$NiSi_2$**	**$1\cdot10^{-7}$**	**(p+) $1\cdot10^{20}$**	**20**	[45]
$NiSi_2$	1-$2.5\cdot10^{-6}$	(n+) > 10^{20}	5	[46]
$NiSi_2$	5-$35\cdot10^{-8}$	(p+) > 10^{20}	5	[46]

Supporting information Figure S14. Nanostructured TEM lamella preparation

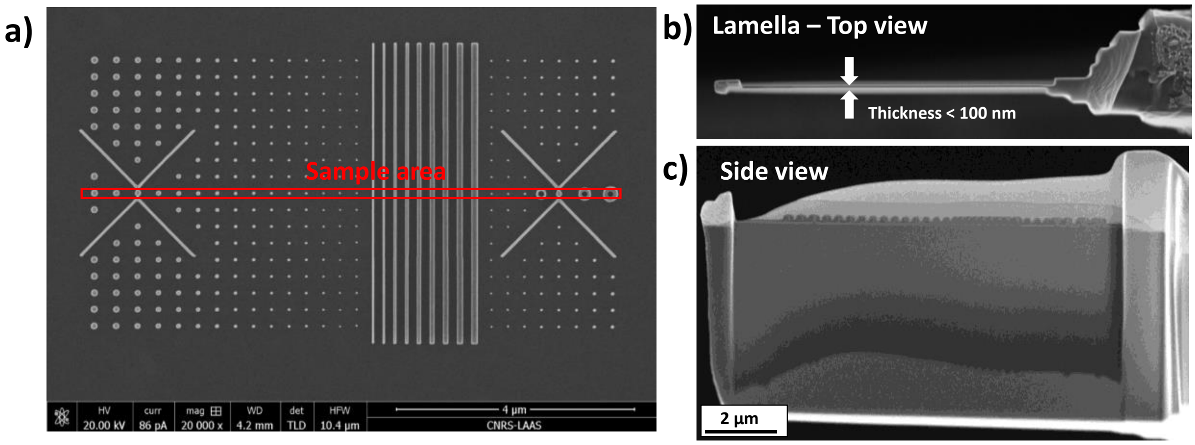

Figure S14. *SEM image of fabricated arrays of NWs and NS after the fabrication of nanostructured silicide contacts (a, top view). Two cross-shaped alignment marks are used to cut out a thin TEM lamella across the entire array. The cut-out lamella is thinned down to less than 100 nm thickness for all samples (b). A finished lamella exhibiting the Si substrate and nanostructure, carbon contrast layer and protective Pt-layers is shown in (c).*

References

[1] Yoshida, M. and Saito, K., Dissociative Diffusion of Nickel in Silicon and Self-Diffusion of Silicon. *Japanese Journal of Applied Physics*, **6** (5), p. 573, 1967. **DOI:** 10.1143/JJAP.6.573

[2] Bonzel, H. P., Diffusion of Nickel in Silicon. *Physica status solidi b*, **20** (2), pp. 493-504, 1967. **DOI:** 10.1002/pssb.19670200210

[3] Yarykin, N., Weber, J., Evidence for room-temperature in-diffusion of nickel into silicon. *Appl. Phys. Lett.*, **109**, p. 102101, 2016. **DOI:** 10.1063/1.4962394

[4] Lindroos, J.,Fenning, D. P.,Backlund, D. J., Verlage, E., Gorgulla, A., Estreicher, S. K., Savin, H., and Buonassisi, T., Nickel: A very fast diffuser in silicon. *J. Appl. Phys.*, **113**, p. 204906, 2013. **DOI:** 10.1063/1.4807799

[5] Tinani, M., Mueller, A., Gao, Y., Irene, E. A., Hu, Y. Z. and Tay, S. P., In situ real-time studies of nickel silicide phase formation. *Journal of Vacuum Science & Technology B: Microelectronics and Nanometer Structures*, **19** (2), p. 376, 2001. **DOI:** 10.1116/1.1347046

[6] Lavoie, C., d'Heurle, F. M., Detavernier, C. and Cabral Jr., C., Towards implementation of a nickel silicide process for CMOS technologies. *Microelectronic Engineering*, **70** (2-4), pp. 144-157, 2003. **DOI:** 10.1016 /S0167-9317(03)00380-0

[7] Chen, L. J., Silicide formation. In Silicide Technology for integrated Circuits, London, United Kingdom: The Institution of Electrical Engineers, 2004. **ISBN:** 0-86341-352-8

[8] Do, P. H., Development of nickel silicide for integrated circuit technology, Master's thesis, Rochester Institute of Technology, Rochester, United States, 2006.
URL: https://scholarworks.rit.edu/cgi/viewcontent.cgi?article=8934&context=theses (Last accessed 06/8/2024)

[9] Massalski, T. B., Subramanian, P. R., Okamoto, H. and Kacprzak, L., Binary Alloy Phase Diagrams, second ed., 1990. **DOI:** 10.1002/adma.19910031215

[10] Tous, L., van Dorp, D. H., Russell, R., Das, J., Aleman, M., Bender, H., Meersschaut, J., Opsomer, K., Poortmans, J. and Mertens, R., Electroless nickel deposition and silicide formation for advanced front side metallization of industrial silicon solar cells. *Energy Procedia*, **21**, pp. 39-46, 2012. **DOI:** 10.1016/j.egypro.2012.05.006

[11] Lavoie, C., Purtell, R., C Coia, C., Detavernier, C., Desjardins, P., Jordan-Sweet, J., Cabral, C., d'Heurle, H. M., and Harper, J. M. E., In situ monitoring of thin film reactions during rapid thermal annealing: Nickel silicide formation. *Rapid Thermal and Other Short-Time Processing Technologies III, Proceedings,* **2002** (11), p.455-467, 2002. **URL:** http://hdl.handle.net/1854/LU-407051 (Last accessed 06/08/2024)

[12] Donoso, C. J., Jay, A., Lam, J., Müller, J., Larrieu, G., Bongiorno, C., La Magna, A., Alberti, A. and Hémeryck, A., A comprehensive atomistic picture of the as-deposited Ni-Si interface before thermal silicidation process. *Applied Surface Science*, **631**, p. 157563 2023. **DOI:** 10.1016/j.apsusc.2023.157563

[13] Rivero, C., Gergaud, P., Gailhanou, M., Thomas, O., Froment, B., Jaouen, H. and Carron, V., Combined synchrotron x-ray diffraction and wafer curvature measurements during Ni–Si reactive film formation. *Applied Physics Letters*, **87** (4), p. 041904, 2005. **DOI:** 10.1063/1.1999021

[14] Khan, M. B., Deb, D., Kerbusch, J. Fuchs, F., Löffler, M., Banerjee, S., Mühle, U., Weber, W. M., Gemming, S., Schuster, J., Erbe, A. and Georgiev, Y. M., Towards Reconfigurable Electronics: Silicidation of Top-Down Fabricated Silicon Nanowires. *Applied Sciences*, **9** (17), p. 3462, 2019. **DOI:** 10.3390/app9173462

[15] Khan, M. B., Prucnal, S., Ghosh, S., Deb, D., Hübner, R., Pohl, D., Rebohle, L., Mikolajick, T., Erbe, A. and Georgiev, Y. M., Controlled Silicidation of Silicon Nanowires Using Flash Lamp Annealing. *Langmuir*, **37** (49), p. 14284-14291, 2021. **DOI:** 10.1021/acs.langmuir.1c01862

[16] Hsu, H.-F., Huang, W.-R., Chen, T.-H., Wu, H.-Y. and Chen, C.-A., Fabrication of Ni-silicide/Si heterostructured nanowire arrays by glancing angle deposition and solid state reaction. *Nanoscale Research Letters*, **8** (224), 2013. **DOI:** 10.1186/1556-276X-8-224

[17] Simon, M., Mizuta, R., Fan, Y., Tahn, A., Pohl, D., Trommer, J., Hofmann, S., Mikolajick, T. and Weber, W. M., Lateral Extensions to Nanowires for Controlling Nickel Silicidation Kinetics: Improving Contact Uniformity of Nanoelectronic Devices. *ACS Applied Nano Materials*, **4** (5), pp. 4371-4378, 2021. **DOI:** 10.1021/acsanm.0c03072

[18] Gösele, U. and Tu, K. N., Growth kinetics of planar binary diffusion couples: ''Thin-film case'' versus ''bulk cases''. *Journal of Applied Physics*, **53** (4), pp. 3252-3260, 1982. **DOI:** 10.1063/1.331028

[19] Nakatsuka, O., Okubo, K., Tsuchiya, Y., Sakai, A., Zaima, S., and Yasudao, Y., Low-Temperature Formation of Epitaxial NiSi2 Layers with Solid-Phase Reaction in Ni/Ti/Si(001) Systems. *Jpn. J. Appl. Phys.*, **44**, p. 2945, 2005. **DOI:** 10.1143/JJAP.44.2945

[20] Thron, A. M., Greene, P. K., Liu, K. and van Benthem, K., Structural changes during the reaction of Ni thin films with (100) silicon substrates. *Acta Materialia*, **60** (6-7), p. 2668-2678, 2012. **DOI:** 10.1016/j.actamat.2012.01.033

[21] Ikarashi, N., Atomic structure of a Ni diffused Si (001) surface layer: Precursor to formation of NiSi2 at low temperature. *Journal of Applied Physics*, **107** (3), p. 033505, 2010. **DOI:** 10.1063/1.3294691

[22] Jiang, Y., Ru, G.-P., Liu, J.-H., Qu, X.-P. and Li, B.-Z., The Reaction Characteristics of Ultra-Thin Ni Films on Undoped and Doped Si (100). *Journal of Electronic Materials*, **33** (7), 2004. **DOI:** 10.1007/s11664-004-0239-y

[23] Tomita, R., A study on formation of high resistivity phases of nickel silicide at small area and its solution for scaled CMOS devices, PhD thesis, Tokyo Institute of Technology, Tokyo, Japan, 2013.
Link: http://www.iwailab.ep.titech.ac.jp/pdf/201303dthesis/tomita.pdf (Last accessed: 06/08/2024)

[24] Tamura, Y., A novel interface controlled silicidation process for future 3D Schottky devices, Master's thesis, Tokyo Institute of Technology, Tokyo, Japan, 2013.
Link: http://www.iwailab.ep.titech.ac.jp/pdf/201303mthesis/tamura.pdf (Last accessed: 06/08/2024)

[25] Brandes, E. A. and Brook, G. B. (Ed.), Smithells Metals Reference Book, 7. Edition, Butterworth-Heinemann, 1988. **ISBN:** 978-0750636247

[26] Song, Y. and Jin, S., Synthesis and properties of single-crystal beta3-Ni3Si nanowires. *Applied Physics Letters*, **90** (17), p. 173122, 2007. **DOI:** 10.1063/1.2732828

[27] Colgan, E. G., Mäenpää, M., Finetti, M. and Nicolet, M.-A., 1983. Electrical characteristics of thin Ni2Si, NiSi, and NiSi2 layers grown on silicon. *Journal of Electronic Materials*, **12** (2), p. 413-422, 1983. **DOI:** 10.1007/bf02651140

[28] Samsonov, G., Vinitskii, I. M., Handbook of Refractory compounds, Springer, 1980. **ISBN:** 978-0306651816

[29] Maex, K. and van Rossum, M., Properties of metal silicides, INSPEC, 1988. **ISBN:** 0 85296 859 0

[30] Mangelinck, D., Mechanisms of Silicide Formation by Reactive Diffusion in Thin Films. *Diffusion Foundations*, **21**, pp. 1-28, 2019. **DOI:** 10.4028/www.scientific.net/df.21.1

[31] Gregoire, M., Silicides in microelectronics: phase sequence, nanoscale effect, and degradation mechanisms. *HDR*, 2022. **URL:** https://hal.science/tel-04193978/ (Last accessed 6/08/2024)

[32] Data retrieved from the Materials Project for (mp-23, mp-507, mp-1118, mp-351, mp-149) from database version v2021.11.10. **URL:** https://materialsproject.org/ (Last accessed: 13/12/2022)

[33] Data retrieved from the PAULING FILE Multinaries database (Edition – 2012) for (sd_0452327). Version 2016.10. **URL:** https://materials.springer.com/isp/crystallographic/docs/sd_0452327 (Last accessed: 13/12/2022)

[34] Yamaguchi, Y., Yoshida, M., and Aoki, H., Solid Solubility of Nickel in Silicon Determined by Use of 63Ni as a Tracer. *Japanese Journal of Applied Physics*, **2** (11), p. 714, 1963. **DOI:** 10.1143/JJAP.2.714

[35] Valeri, S., Del Pennino, U. and Sassaroli, P., Oxidation behaviour of nickel silicides investigated by AES and XPS. *Surface Science Letters*, **134** (3), p. L537-L542 1983. **DOI:** 10.1016/0167-2584(83)90632-1

[36] D'Heurle, F. M., The Oxidation of Silicides on Silicon. In *The Physics and Chemistry of SiO2 and the Si-SiO2 Interface*, p. 85-94, 1988. **DOI:** 10.1007/978-1-4899-0774-5_9

[37] Wang, T., Guo, Q., Liu, Y. and Yun, J., Abnormal oxidation in nickel silicide and nickel germanosilicide in sub-micron CMOS. *Chinese Physics B*, **21** (6), p. 068502, 2012. **DOI:** 10.1088/1674-1056/21/6/068502

[38] Rahman, M. K., Nemouchi, F., Chevolleau, T., Gergaud, P. and Yckache, K., Ni and Ti silicide oxidation for CMOS applications investigated by XRD, XPS and FPP. *Materials Science in Semiconductor Processing*, **71**, p. 470-476, 2017. **DOI:** 10.1016/j.mssp.2017.06.025

[39] Verleysen E., Bender H., Richard O., Schryvers D. and Vandervorst W., Compositional characterization of nickel silicides by HAADF-STEM imaging. *Journal of Materials Science*, **46**, pp. 2001-2008, 2011. **DOI:** 10.1007/s10853-010-5191-z

[40] Klinger, M. and Jäger, A., Crystallographic Tool Box (CrysTBox): automated tools for transmission electron microscopists and crystallographers. *Journal of Applied Crystallography*, **48** (6), 2015. **DOI:** 10.1107/S1600576715017252.

[41] Geuens, P, and Van Dyck, D., About forbidden and weak reflections. *Micron*, **34** (3–5), pp. 167-171, 2003. **DOI:** 10.1016/S0968-4328(03)00032-5

[42] Thron, A. M., Greene, P. K., Liu, K. and van Benthem, K., Structural changes during the reaction of Ni thin films with (100) silicon substrates. *Acta Materialia*, **60** (6-7), p. 2668-2678, 2012. **DOI:** 10.1016/j.actamat.2012.01.033

[43] *Data retrieved from the Materials Project for Si2Ni (mp-2291)* and *for Si (mp-149) from database version v2023.11.1.*

[44] Pascu, R., and Romanitan, C., Phase transition of nickel silicide compounds and their electrical properties. *J Mater Sci: Mater Electron*, **32**, pp. 16811–16823, 2021. **DOI:** 10.1007/s10854-021-06238-1

[45] Zaimaa, S., Nakatsukab, O., Sakaic, A., Murotad, J., and Yasuda, Y., Interfacial reaction and electrical properties in Ni/Si and Ni/SiGe(C) contacts. *Applied Surface Science*, **224**, pp. 215–221, 2004. **DOI:** 10.1016/j.apsusc.2003.08.049

[46] Zhao, Q.-T., Knoll, L., Zhang, B., Buca, D., Hartmann, J.-M., and Mantl, S., Ultrathin epitaxial Ni-silicide contacts on (100) Si and SiGe: Structural and electrical investigations, *Microelectronic Engineering*, **107**, pp. 190-195, 2013. **DOI:** 10.1016/j.mee.2012.10.014.

[47] Chen, S.-Y., Yeh, P.-H., Wu, W.-W., Chen, U.-S., Chueh, Y.-L., Yang, Y.-C., Gwo, S., and Chen, L.-J., Low Resistivity Metal Silicide Nanowires with Extraordinarily High Aspect Ratio for Future Nanoelectronic Devices. *ACS Nano*, **5** (11), pp. 9202-9207, 2011. **DOI:** 10.1021/nn203445p

[48] Stavitski, N., van Dal, M. J. H., Lauwers, A., Vrancken, C., Kovalgin, A. Y., Wolters, R. A. M., Systematic TLM Measurements of NiSi and PtSi Specific Contact Resistance to n- and p-Type Si in a Broad Doping Range. *IEEE Electron Device Letters*, **29** (4), 2008. **DOI:** 10.1109/LED.2008.917934
